# Rationalizing Decision-Making: Understanding the Cost and Perception of Time

**DOI:** 10.1163/24054496-00101004

**Published:** 2014-01-01

**Authors:** Vijay Mohan K. Namboodiri, Stefan Mihalas, Marshall G. H. Shuler

**Affiliations:** 1 The Johns Hopkins University, Department of Neuroscience, Baltimore, MD; 2 The Allen Institute for Brain Science, Seattle, WA

**Keywords:** intertemporal decision-making, time, time perception, temporal discounting, subjective value, impulsivity, Optimal Foraging Theory, Ecological Rationality Theory, hyperbolic discounting, Discounted Utility Theory, Scalar Expectancy Theory, Behavioral Theory of Timing, Weber’s law, Training-Integrated Maximized Estimation of Reinforcement Rate

## Abstract

Humans, as with other animals, decide between courses of action based on the evaluation of the relative worth of expected outcomes. How outcome magnitude interacts with temporal delay, however, has yet eluded a principled understanding that reconciles the breadth of well-established behaviors in intertemporal decision-making. Here, we review the history of this endeavor to rationalize decision-making regarding the domain of time, highlighting extant theories, their limitations, and recent experimental and theoretical advances. These new advances recast long presumed deficiencies in observed decision-making behavior, not as flaws, but rather as signs of optimal decision-making under experiential constraints. This new conception naturally unites the fields of intertemporal decision-making and time perception, which have long been recognized to be interconnected but not yet unified in a formal framework.

## 1 Introduction

Humans and other animals have evolved to accumulate food and other rewards like water, sex, wealth, etc. Frequently, rewards are available only as a result of deliberate actions in their pursuit. For instance, a hungry lion might have to decide between two areas of the forest for foraging, one closer but with fewer prey and the other farther but with more prey. In order to be successful in the wild, animals must have evolved an effective mechanism to make such complex decisions, comparing between multiple options with differing magnitudes, delays and probabilities of rewards. Humans, too, routinely make such decisions in their day-to-day lives, to choose, for instance, between a closer but less preferred coffee shop and a farther, but better one. The question of how animals, including humans, make such intertemporal decisions has been the subject of at least eight decades of active research, in fields as diverse as economics, psychology, evolutionary ecology, neuroscience and addiction. Across the spectrum of these fields, researchers have approached this problem in myriad ways, with some proposing theories of animal behavior and others measuring animal behavior experimentally. However, there has not yet been significant agreement between theories and experiments. Here, we review theoretical work addressing this problem, in the context of recent advances in reconciling theories with experiments. For the purpose of this review, we focus only on a subset of the general problem, in the dimension of time, ignoring how differing probabilities and risks affect decisions.

In order to make decisions about delayed rewards, animals must be able to measure those delays. Hence, the problem of “intertemporal decision-making” is intertwined with time perception. However, theoretical and experimental work on 1) how animals measure delays, and, 2) how they make decisions between differently delayed rewards, has been largely non-overlapping. Nevertheless, there has been some recent work, both theoretical and experimental, that examines the connection between how animals make intertemporal decisions and how they perceive time. In the latter half of the review, we therefore focus on time perception, in the context of recent theoretical and experimental attempts to create a unified understanding of intertemporal decision-making and time perception.

First, we will provide a historical account of theories of intertemporal decision-making.

### 1.1 History of Theories of Intertemporal Decision-Making

The problem of intertemporal choice was first mentioned in 1834 by John Rae (Rae, 1834). In his work, he wondered about the origins of differences in wealth between nations. Rae claimed that one of the key determinants of wealth of a nation is the nation’s “desire of accumulation”. The desire of accumulation, according to him, was determined by the balance between two kinds of psychological factors, one that motivates the nation to invest and save for the future and another that derives pleasure from immediate consumption. Thus, the idea of making decisions about delayed outcomes was framed as the conflict between two different psychological impulses of humans (and societies).

The notion of intertemporal choices being driven by innate psychological quantities stuck ever since John Rae and persists to this day (e.g., Bickel et al., 2007; Frederick et al., 2002; Kalenscher & Pennartz, 2008, Madden & Bickel, 2010; McClure et al., 2004; van den Bos & McClure, 2013). The first attempt to mathematically formalize the treatment of such psychological factors was made a century later by Paul Samuelson in 1937 (Samuelson, 1937, but see Bohm-Bawerk, 1889; Fisher, 1930) when he invented the “Discounted Utility Theory” (DUT). It is important to point out that DUT was borne out of ideas that originated from John Rae, and thus shared many of the same limitations. Nevertheless, it was the first simple and mathematically tractable formulation of the problem of intertemporal choice. Briefly, DUT states that intertemporal choices are made so as to maximize the “net discounted utility” of the future. Here, DUT assumes that the “utility” of a reward is given by its face value, if it were to be received immediately. For instance, the utility of $20 is $20, irrespective of when you receive it. However, the immediate subjective value of $20 delayed by a fixed amount would be given by its “discounted utility”.

The key postulate of DUT is that the “discounted utility” of a delayed reward is determined by an exponential discounting function, with the exponential constant—the discount rate—determining the ability of a person to delay gratification. In simpler terms, the discount rate measures the patience of an individual—the lower the discount rate, the higher your patience in waiting for a reward, i.e. the longer you are willing to wait to obtain that reward. The major simplification achieved by Samuelson was in compressing the different psychological factors of John Rae and others into a single, measurable parameter of self-control.

The other major advance made by Samuelson was in treating the utility of different future rewards as the sum of their respective discounted utilities. This “net discounted utility”, as mentioned earlier, was expressed as shown below.

$$\text{Maximize}\;U=\int_{0}^{T}D(t)u(t)dt;\;\text{where}\;D(t)=(1)$$

Here, D(t) is the discounting function, and u(t) is the utility of a single reward received at a delay of t. Time was assumed to be integrated up to a maximum delay of consideration, also known as the temporal horizon of a decision, represented by T.

Samuelson’s model was simple and elegant, with its exponential form particularly attractive, owing to similarities with the calculation of compound interest. It further provided a single, measurable parameter of an individual’s ability to delay gratification (viz. k). Despite all these advantages, however, Samuelson had a number of reservations about the validity and utility of his model. Since most of these concerns were ignored by future researchers due to its distinct advantages, we would like to point them out here, in Samuelson’s own words.

#### Concern 1:

“In the first place, it is completely arbitrary to assume that the individual behaves so as to maximize an integral of the form envisaged. This involves the assumption that at every instant of time the individual’s satisfaction depends only upon the consumption at that time, and that, furthermore, the individual tries to maximize the sum of instantaneous satisfactions reduced to some comparable base by time discount.”

#### Concern 2:

“A less important point to be noted is the fact that our equations hold only for an individual who is deciding at the beginning of the period how he will allocate his expenditures over the period. Actually, however, as the individual moves along in time there is a sort of perspective phenomenon in that his view of the future in relation to his instantaneous time position remains invariant, rather than his evaluation of any particular year (e.g. I940). This relativity effect is expressed in the behavior of men who make irrevocable trusts, in the taking out of life insurance as a compulsory savings measure, etc….Moreover, in the analysis of the supply of savings, it is extremely doubtful whether we can learn much from considering such an economic man, whose tastes remain unchanged, who seeks to maximize some functional of consumption alone, in a perfect world, where all things are certain and synchronized.”

In sum, Samuelson wrote *“any connection between utility as discussed here and any welfare concept is disavowed*”, stressing that there was no *a priori* reason why his model would be a valid or normative way of describing human behavior.

Despite these strong reservations, Samuelson’s work paved the way for the exponential model of discounting, and more generally, the idea of discounting functions, to be ingrained into work on intertemporal choices (Benzion et al., 1989; Fehr, 2002; Fishburn & Rubinstein, 1982; Frederick et al., 2002; Kalenscher & Pennartz, 2008; Laibson, 1997; Lancaster, 1963; Madden & Bickel, 2010; Thaler, 1981; van den Bos & McClure, 2013). DUT received further theoretical support in 1960 when Koopmans showed that DUT can be derived from a set of simple axioms (Koopmans, 1960). The most important axiom was the statement (one that Samuelson recognized, as mentioned above) that the intertemporal preferences of an individual are stationary over time, i.e. if an individual prefers option 1 in a choice between option 1 and option 2, if the delay between the two options is fixed, the individual will always prefer option 1, independent of how much time has elapsed since the first decision was made. Like Samuelson, Koopmans also did not argue for the normative or descriptive validity of these axioms.

Nevertheless, further work on intertemporal choice in economics considered consistent time preferences and stationarity as a fundamental tenet of human rationality (see Drouhin, 2009; Fishburn & Rubinstein, 1982; Frederick et al., 2002; Koopmans, 1960; Lapied & Renault, 2012a, 2012b; Strotz, 1956 for a discussion). However, experimental evidence repeatedly showed that this core postulate is violated by humans (Ainslie, 1975; Ainslie & Monterosso, 2003; Benzion et al., 1989; Frederick et al., 2002; Green et al., 1994; Holt et al., 2003; Loewenstein & Prelec, 1992; McClure et al., 2004; Thaler, 1981), pigeons (Ainslie, 1974; Chung & Herrnstein, 1967; Rachlin et al., 1972) and rats (Bennett, 2002; Ito & Asaki, 1982). In fact, the axiom of constant time preference can be immediately seen to be violated by considering the following two example choices: “which would you prefer: $100 now or $105 in a month?” and “which would you prefer: $100 in a year or $105 in a year and one month?” It should be immediately clear that while most people prefer $105 in the second question, they prefer $100 in the first, thus violating the assumption of stationary preferences.

In the face of such overwhelming evidence, there are two possible recourses to solving the apparent contradiction: 1) change one’s definition of rationality, or, 2) state that all the animals tested above are irrational. Many economists chose the latter option, maintaining the axiom of stationary time preferences (and exponential discounting) as “rational” (see Drouhin, 2009; Lapied & Renault, 2012a, 2012b for a discussion). This is partly due to equating stationarity and time consistency (see Drouhin, 2009; Lapied & Renault, 2012a, 2012b for a discussion), and partly due to the fact that other models of rationality, like reward rate maximization (Bateson, 2003; Bateson & Kacelnik, 1996; Stephens & Anderson, 2001; Stephens & Krebs, 1986; Stephens, 2008), have not been successful in explaining animal behavior.

In addition to the violation of the assumption of stationarity of choices, many other assumptions of DUT have been shown to be violated by human behavior. For instance, DUT assumes consumption independence—the utility of a reward does not depend on whether or not that reward was obtained in the immediate past. This is patently false, as surely animals can become sated over time. Consider too, for instance, that the preference for a restaurant will obviously depend on whether or not an individual ate there for the past five days. There are numerous other violations of DUT that are not going to be discussed here. For a more detailed overview of these violations, see prior reviews on this topic (Frederick et al., 2002; Kalenscher & Pennartz, 2008).

An assumption related to the stationarity axiom is that the discount factor (rate of discounting per unit discounting) is constant over time. Behavioral scientists and psychologists have shown that this, too, is repeatedly violated in experiments across many different species (Ainslie, 1974, 1975; Ainslie & Monterosso, 2003; Benzion et al., 1989; Calvert et al., 2010; Green et al., 1994; Holt et al., 2003; Kobayashi & Schultz, 2008; Rachlin et al., 1972; Thaler, 1981). In fact, it was observed experimentally in 1967 that delayed food rewards were preferred in inverse proportion to their delay (Chung & Herrnstein, 1967). This relationship was later mathematically reformulated within the framework of a discounting function by Ainslie (Ainslie, 1975) and experimentally confirmed by numerous subsequent papers (e.g., Ainslie, 1974, 1975; Ainslie & Monterosso, 2003; Benzion et al., 1989; Calvert et al., 2010; Green et al., 1994; Holt et al., 2003; Kobayashi & Schultz, 2008; Rachlin et al., 1972; Thaler, 1981). Specifically, this discounting function, that approximated intertemporal choice behavior better than exponential discounting functions, was hyperbolic in form. The animal’s choice under this conception can be expressed mathematically as shown below:

(2) $$\mathrm{Choose}\;max(r(t)D(t));D(t)=\frac{1}{1\;+\;kt}$$

where D(t) represents the discounting function, r(t) is the reward magnitude of a reward available at delay t and k is the discounting constant.

It is important to point out that the casting of experimental observations into a “discounting function” framework is due to the legacy of Samuelson’s work (Samuelson, 1937). In this view, the agency for the reduction in value of a reward with delay (temporal discounting) is due completely to a psychologically-innate discounting function. The contribution of Ainslie provided a much better descriptive model of experimental data. However, the contribution was only to find that a hyperbolic discounting function provided a better fit to experimental data than an exponential discounting function, and not a rationale for *why* hyperbolic discounting would better describe the data.

Recently, many other forms of discounting functions have been proposed that provide even better fits to experimental data than pure hyperbolic discounting functions (al-Nowaihi & Dhami, 2008; Green & Myerson, 2004; Killeen, 2009; Laibson, 1997; McClure et al., 2004; Schweighofer et al., 2006; van den Bos & McClure, 2013). Among the most prominent are quasi-hyperbolic discounting functions (Laibson, 1997) and β-δ discounting functions (McClure et al., 2004; van den Bos & McClure, 2013).

A major limitation of the hunt for the perfect discounting function to fit experimental data is that it provides only that—a fit to the data. It does not provide an explanation for why animals discount delayed rewards the way they do. Further, it also cannot rationalize the observed steepness of temporal discounting (a measure of patience, like k in DUT) of an individual in a given reward environment. Hence, all the different discounting functions described above are fits to the data which are assumed to originate from some innate psychological quality. For instance, finding that β -δ discounting functions provide better fits to experimental data has been used to infer that the brain has two separate systems involved in processing immediate and delayed rewards, respectively. Indeed, recent human imaging studies have shown that different areas of the brain are differentially involved when a subject is considering an immediate or a delayed reward (McClure et al., 2004, see Kalenscher & Pennartz, 2008; van den Bos & McClure, 2013 for reviews).

A wholly different perspective to the problem of intertemporal choice, not wed to the notion of a discounting function, can be obtained by considering the field of behavioral ecology. In 1966, a highly influential paper was published in theoretical behavioral ecology by MacArthur and Pianka (MacArthur & Pianka, 1966). This paper considered how foraging animals should decide between different patches of food, if they behaved economically. It introduced the idea that animals should forage so as to maximize their net energy intake in the long run. Net energy intake was operationally defined as the total reward obtained per unit time spent in obtaining it. The idea behind the postulate was simple: obtaining a maximal rate of food intake would maximize the chances of living. Hence, in this conception, time was not merely a component of decision-making, but the most important dimension over which rewards needed to be accumulated.

The idea of maximizing long-term reward rates was the key thesis behind many subsequent theories on optimal foraging (Charnov, 1976a, 1976b; Krebs, 1978; Krebs et al., 1977; Pyke et al., 1977; Pyke, 1984). This literature is too vast to be fully considered here. For a detailed review of such theories, see Pyke (1984), and Stephens and Krebs (1986). Together, these theories came to be known as the Optimal Foraging Theory (OFT).

Mathematically, OFT’s postulate of maximizing long-term reward rates can be expressed as:

(3) $$Maximize\frac{\sum_{i=1}^{\infty }r_{i}}{\sum_{i=1}^{\infty }t_{i}}$$

Here, all possible future rewards are added in the numerator ($r_{i}$ is an individual reward) and divided by the total time it takes to acquire them. $t_{i}$ is the delay in getting the $i^{\mathrm{th}}$ reward from the moment that the $(i-1{)}^{\mathrm{th}}$ reward was obtained. If the sum is carried over to infinity as shown above, the total time spent as shown in the denominator will be ∞. A more realistic conception would be to state that the long term reward rate is calculated not over an infinite future time horizon, but a finite (though appreciable), time horizon, T, similar to the temporal horizon considered in equation (1) for DUT. Thus, equation (3) can be rewritten as:

(4) $$\mathrm{Maximize}\frac{\int_{0}^{T}r(t)dt}{T}$$

where r(t) is the reward available at a delay of t. This equation looks similar in form to equation (1) of DUT, but with a key difference: the quantity that is being maximized is not the net discounted utility, but the net reward rate.

Let us now consider how equation (4) was used to predict the intertemporal decisions of animals. Consider an animal choosing between two reward options, ($r_{1},t_{1}$) and $\left(r_{2},t_{2}\right)$, where the ordered pair represents the reward magnitude and delay to reward, respectively. Consider too that after either reward, the animal has to wait a fixed intertrial interval (ITI) until foraging again. This might represent the time required to consume the rewards, for instance.

It was argued, then, that the choice of the animal between option 1 and option 2 would be effectively the choice between the long-term reward rate obtained if the animal picked option 1 alone, and, the long-term reward rate obtained if the animal picked option 2 alone. Thus, the animal’s choice could be written as

(5) $$\mathrm{Choose}\;max(\frac{r_{i}}{ITI\;+\;t_{i}});i\in \{1,2\}$$

Here, the total reward rate achieved if the animal picked only option i is the reward magnitude of option i divided by the total effective time spent per receipt of reward.

Interestingly, a closer examination of equation (5), along with equation (2) reveals that the choice of maximizing reward rate as expressed in (5) is similar to maximizing a hyperbolically-discounted reward, with the hyperbolic constant k (Equation (5)) replaced by 1/ITI. Thus, it was argued that maximizing long-term reward rate underlies the experimentally observed hyperbolic discounting function mentioned earlier. This was the first instance wherein the agency for the decay in value of delayed rewards was not placed on an innate discounting function, but on the need to maximize an ecologically-relevant metric of fitness, i.e. reward rate.

Especially in the context of foraging, maximizing fitness or reward rate was a better definition of “rational” decision-making than stationarity of time preferences. However, it was soon obvious that while the experimental data on intertemporal decisions indicated a hyperbolic discounting function, this function did not result from a maximization of reward rates as considered in OFT. This was because, in experiments in which animals were given choices between two reward options but where the total trial duration was a constant (and not the ITI), animals did not always pick the larger reward (e.g., Ainslie, 1974; Bateson & Kacelnik, 1996; Blanchard et al., 2013; Cardinal et al., 2001; Grossbard & Mazur, 1986; Kacelnik & Bateson, 1996; Kalenscher et al., 2005; Kobayashi & Schultz, 2008; Louie & Glimcher, 2010; Mazur, 1988; Pearson et al., 2010; Rachlin et al., 1972; Roesch et al., 2007; Stephens & Anderson, 2001; Winstanley et al., 2004). This was a direct challenge to the choice algorithm shown in equation (5) since the effective time spent on either option was equal, but the animals still did not pick the option with the larger reward magnitude.

In the face of apparent empirical rejection, behavioral ecologists proposed an alternative decision-making algorithm within the realm of rate maximization. It was proposed that instead of maximizing long-term reward rates, animals maximized single-trial reward rates (Bateson & Kacelnik, 1996; Real, 1991; Stephens et al., 2004; Stephens & Krebs, 1986; Stephens, 2008). This approach has been referred to by many different names across the literature (Bateson & Kacelnik, 1996; Real, 1991; Stephens et al., 2004; Stephens & Krebs, 1986; Stephens, 2008), but we will refer to it by the name of Ecological Rationality Theory (ERT), as used by Stephens et al. (2004) and Stephens (2008). Regardless of the name, however, all these papers propose the following choice algorithm:

(6) $$\mathrm{Choose}\;max(\frac{r_{i}}{t_{i}})$$

The major difference between the predictions of equations (5) and (6) is in the inclusion/exclusion of the various delay components into the decision. While equation (5) (OFT) considers all effective intervals within a trial, ERT only considers the delay to reward. Post-reward delays of any sort are excluded in the decision algorithm of ERT. The rationale for their exclusion was that in the wild, foraging animals rarely decide between options with differing post-reward delays associated with them. In fact, it was argued that typical decisions in the wild are not even in the form of binary choices as presented above. Instead, it was argued, that typical decisions in the wild have a “foreground-background nature”, i.e. that a choice is presented always in the context of background activity. Interestingly, it was shown that in laboratory tasks where such a foreground-background structure was presented to animals, equation (6) resulted in long-term reward rate maximization, as expressed in equation (4) (Stephens & Anderson, 2001; Stephens, 2008). This result—that in typical foraging decisions, maximizing single-trial reward rates is the same as maximizing long-term reward rates—provided support for the argument that animals only maximized single-trial reward rates, and is the basis of the name, Ecological Rationality Theory.

While ERT provided better fits to experimental data in the foraging literature than OFT (e.g., Bateson & Kacelnik, 1996; Stephens, 2008; Stephens & Anderson, 2001), it was nevertheless insufficient at providing quantitative fits to the experimental data collected in standard psychological tasks involving binary choices. This was because the effective discounting constant predicted by equation (6), according to a hyperbolic discounting function, is ∞. This is immediately apparent if one realizes that equation (5) (where k=1/ITI reduces to equation (6) when ITI=∞, for when the ITI is infinite, the decision is effectively only over one trial. Yet, every discounting study measured finite, non-zero discounting constants (Ainslie, 1974; Bateson & Kacelnik, 1996; Blanchard et al., 2013; Cardinal et al., 2001; Grossbard & Mazur, 1986; Kacelnik & Bateson, 1996; Kalenscher et al., 2005; Kobayashi & Schultz, 2008; Louie & Glimcher, 2010; Mazur, 1988; Pearson et al., 2010; Rachlin et al., 1972; Roesch et al., 2007; Stephens & Anderson, 2001; Winstanley et al., 2004).

In conclusion, theories from behavioral ecology that attempted to rationalize intertemporal decision-making within the framework of reward-rate maximization failed to provide satisfactory explanations for empirical data. Further, the theory (DUT) based on the assumption that temporal discounting must lead to stationary time preferences, and hence should abide by exponential discounting, is also inconsistent with experimental observations. Only psychological models like the hyperbolic discounting model proposed by Ainslie (1974, 1975), among others (e.g., al-Nowaihi & Dhami, 2008; Green & Myerson, 2004; Killeen, 2009; Laibson, 1997; McClure et al., 2004; Schweighofer et al., 2006; van den Bos & McClure, 2013), provided good fits to observed data from laboratory tasks. These models, however, are not based on any normative principle of decision-making, unlike DUT, OFT and ERT, and therefore do not rationalize why apparent discounting functions take the shape that they do.

## 2 Recent Experimental and Theoretical Advances in the Study of Intertemporal Decision-Making

In this section of the review, we will focus on recent advances in aligning theories and experiments of intertemporal decision-making. For this purpose, we only consider normative theories and models that propose principles of intertemporal decision-making. For recent descriptive models that attempt to fit data, please refer to the following literature: al-Nowaihi and Dhami (2008), Killeen (2009), and van den Bos and McClure (2013).

### 2.1 Experimental Advances

As was mentioned in Section 1.1, the crucial observation that invalidated the idea of long-term reward rate maximization was the finding that animals do not include post-reward delays in their decisions (e.g., Ainslie, 1974; Bateson & Kacelnik, 1996; Blanchard et al., 2013; Cardinal et al., 2001; Grossbard & Mazur, 1986; Kacelnik & Bateson, 1996; Kalenscher et al., 2005; Kobayashi & Schultz, 2008; Louie & Glimcher, 2010; Mazur, 1988; Pearson et al., 2010; Rachlin et al., 1972; Roesch et al., 2007; Stephens & Anderson, 2001; Winstanley et al., 2004). This meant that in a choice between two rewards that effectively take the same time, the animals did not necessarily choose the larger reward, hence violating the idea of reward rate maximization. It was also found that delays common to both options, like the ITI term in Equation (5) did not have an effect that matched that expected from maximizing long-term reward rates (e.g., Bateson & Kacelnik, 1996; Logue et al., 1985; Mazur, 1989, 2001; Snyderman, 1987; Stephens & Anderson, 2001).

While these observations were a severe challenge to the idea of long-term reward rate maximization, some researchers realized that it was not a death blow (Blanchard et al., 2013; Kacelnik & Bateson, 1996; Stephens, 2002). An explanation for these observed results could be that animals are not able to learn the association between the rewards and their corresponding post-reward delays. Were they able to learn the association between a post-reward delay and the choices presented, they could potentially have chosen the larger option, consistent with reward rate maximization. Hence, it is not that animals do not maximize reward rates; it is just that they do so within constraints of evolved mechanisms of associative learning. This idea was first presented in 1996 by Alex Kacelnik and Melissa Bateson (Kacelnik & Bateson, 1996). They proposed the following mechanistic explanation for why delays following the receipt of reward might not be learned by animals performing standard intertemporal decision-making tasks.

In order to measure the preferences of animals between two delayed rewards, they are first trained to learn the meaning of two conditioned stimuli (CS) (usually visual or auditory cues) that correspond to either reward option. In other words, prior to receiving the choices, the animals are presented with the CSs and their associated delayed rewards and post-reward delays. Once the animals learn the meaning of both CSs, they are presented with choice trials in which they should pick one or the other CS, so as to receive the corresponding delayed reward. In order to learn the association between a CS and the corresponding reward, it was argued that it makes functional sense for the learning to be driven by the receipt of reward, so as to learn the causal relationship between the CS and the reward (Dickinson, 1980; Kacelnik & Bateson, 1996). In this framework, the retrospective assignment of value to the CS is driven only by the reward and not the moment of expiry of the post-reward delay. Hence, it was argued that the reason for choice behavior being insensitive to post-reward delays was simply due to the lack of its learning. For a similar mechanistic argument from reinforcement-theory, based on neural recordings from honeybees, see Montague et al. (1995).

An indirect support to the above hypothesis was provided in 2010 when Pearson et al. found that presenting explicit cues to indicate the lapse of a post-reward delay made the choice behavior of monkeys move towards long-term reward rate maximization (Pearson et al., 2010). Specifically, they designed the CSs to be vertical bars whose length was proportional to the net delay associated with that choice. The moment of receipt of reward was indicated by a colored horizontal line placed at a location corresponding to the delay to reward. The color of the reward line represented the reward magnitude. Hence, the post-reward delays were indicated by the length of the bar following the reward line. Crucially, as the monkeys chose an option, the other option disappeared from the screen and the chosen option’s vertical bar started to shrink in proportion to the passage of time. In this way, the monkeys were explicitly cued to the passage of the delay to reward and the post-reward delay. Interestingly, the explicit cueing was sufficient to increase the monkeys’ choice for the larger reward, thus indicating that in the presence of explicit information of the post-reward delays, the choice behavior of monkeys accorded better with long-term reward rate maximization.

**Figure 1.**
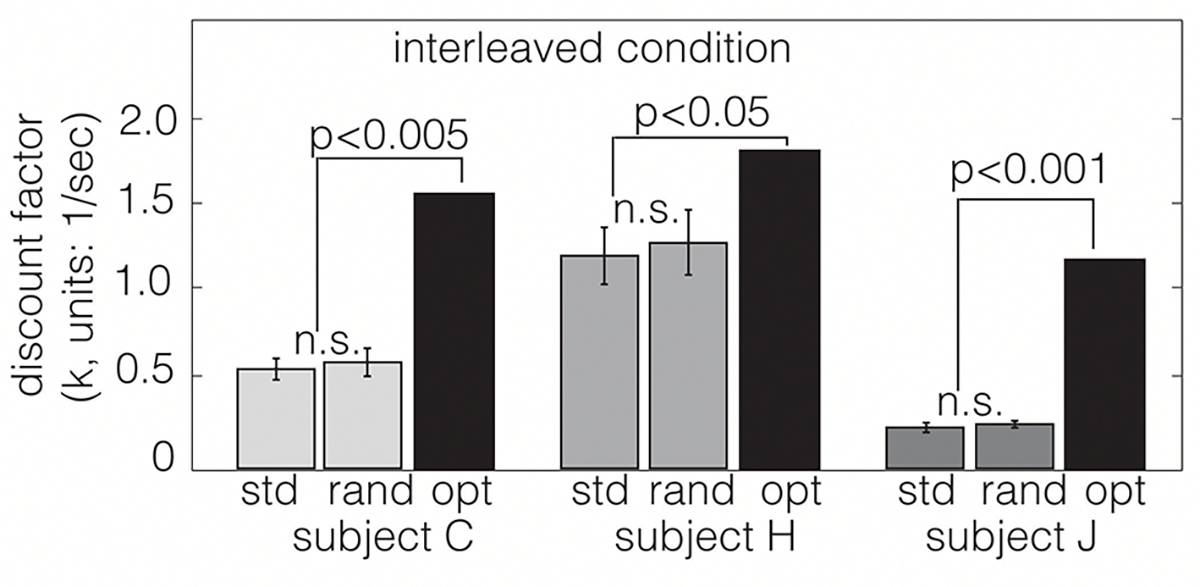
Behavior of 3 monkeys is shown in a task that compares their performance between a standard intertemporal decision-making task (“std”) in which post-reward delays are adjusted to make the trial duration constant, and a random variant of the task in which the post-reward delays are randomly chosen for each option (“rand”). As can be seen, all three monkeys showed similar discounting steepness across the standard and random variant, indicating that they did not appropriately learn the relationship between the rewards and their corresponding post-reward delays. The optimal performance of an agent that correctly learns this relationship is shown by the black bar (“opt”), showing that none of the monkeys are optimal. Adapted from Figure 3 of Blanchard et al. (2013).

Nevertheless, a direct test of the above-mentioned hypothesis would have been to test whether animals performing intertemporal choices can associate the appropriate post-reward delay with a given option. This is exactly what Blanchard and colleagues did in 2013 (Blanchard et al., 2013. In their study, they performed three variants of the experiment mentioned above, but without any explicit cueing of the post-reward delay. In other words, in all their three experiments, the vertical bars only indicated the delay to reward. In the first experiment, they compared the performance of monkeys in the standard intertemporal choice task (with post-reward delays adjusted to have constant trial duration) to their performance in a task where the post-reward delays were randomly shuffled between the options. If the monkeys were able to correctly associate the post-reward delays with their corresponding reward, their performance would be different across these two versions. However, their results clearly indicated that monkeys failed to associate the post-reward delays to their corresponding reward option (Figure 1).

Since the above hypothesis claims that learning is only triggered by the receipt of reward, another prediction is that when the post-reward delays are followed by a reward, they should be included in the decision. Blanchard et al. (2013) confirmed this prediction in a second experiment by presenting a small, but equal volume of reward at the end of the post-reward delays of both options. This, too, increased the likelihood of the monkeys choosing the larger reward.

Finally, in the third experiment, they addressed whether the monkeys’ preferences were sensitive to the magnitude of the post-reward delay. In this experiment, they used an equal post-reward delay for both reward options, much like a constant ITI in earlier experiments, and varied it from 0s to 10s. Their results showed a clear dependence of the discounting steepness on the duration of the post-reward delay. This is similar to the effect of the ITI term in Equation (5), except that the parameter that fit the choice data was always significantly lower than the real delay used. The reason for this discrepancy was claimed by the authors to result from a biased measure of the post-reward delay by the monkeys. However, it must be pointed out that prior tasks studying the effect of intertrial intervals did not observe as clear a dependence of the discounting constant on the ITI (Bateson & Kacelnik, 1996; Logue et al., 1985; Mazur, 1989, 2001; Snyderman, 1987; Stephens & Anderson, 2001). Further, in many timing tasks, animals’ perception of time is much more accurate than is required for these results to be explained by biased time perception, i.e., humans and other animals represent intervals in the msec to minutes range quite accurately (e.g., Allman et al., 2014; Buhusi & Meck, 2005; Matell & Meck, 2004; Merchant et al., 2013). Thus, it is likely that the reason for this discrepancy might not result fully from an inaccurate perception of post-reward delays. For a more detailed discussion of this point, see Section 2.2.

In sum, the Blanchard et al. (2013) experiments convincingly demonstrate that the reason for insensitivity of choice behavior to post-reward delays is the lack of ability of animals to correctly associate those delays with the corresponding reward option. Hence, it would be incorrect to rule out theories of long-term reward rate maximization purely on the basis of this observation. In the next section, we present a novel theory of intertemporal decision-making which is based on reward rate maximization. We present the theory from first principles. A more rigorous treatment of the theory can be found in Namboodiri et al. (2014).

### 2.2 Theoretical Advances

Were decision-making simply the evaluation of offered reward magnitudes, the learning of the relationship between cues and their associated reward amounts would suffice to understand choice behavior. However, if offers are differentially displaced in time, the simple determination of which cue connotes the greatest reward no longer suffices to understand choice behavior should the goal of an animal be to gather the most reward while in an environment. The reason, as previously mentioned, is that time itself has a cost; in having chosen to pursue a larger later reward over a smaller earlier reward, the difference in reward amount achieved must outstrip the difference in the time invested which could otherwise be put to use in further gainful activity.

In the prior sections we reviewed theories that contend with this issue. One such theory, DUT, is based on the normative argument that intertemporal decisions must be stationary in time, which, as mentioned, does not hold up to experimental scrutiny. And why, if mechanisms of intertemporal decision-making evolved under pressures of foraging, would stationary preferences be a better normative argument than simply the maximization of reward rates? From an evolutionary perspective, theories based on reward rate maximization (Stephens & Anderson, 2001; Stephens et al., 2004; Stephens & Krebs, 1986) are more compelling, yet also do not well explain experimental data as animals do not include post-reward delays in their decision-making. Nevertheless, the exclusion of post-reward delays does not in itself rule out rate maximization as the goal, as it has been shown that their disregard is a consequence of an inability to learn the experimental contingency (Blanchard et al., 2013). Hence, our goal here is to approach the problem from the point of view of reward rate maximization, as in OFT. However, whereas OFT only considers future options inferred from the current choice as affecting decision-making, we show that it is the past that matters to estimate the cost of time so as to maximize long-term reward rates.

To appreciate this difference in approach, let us start with the stated goal of OFT—to maximize long-term reward rates. As one cannot change the decisions made in the past, what is wrong with the seemingly reasonable notion in OFT that decision-making to maximize reward rate would be concerned only with the future, being wholly prospective in its outlook? An obvious constraint on so seeking to maximize long-term reward is that, in most decisions, one cannot know the future pattern of rewards beyond those currently available. But for the sake of argument, let us ignore this constraint and consider the case wherein an animal (or an agent) can see many choices (or trials) into the future. Specifically, let us consider an agent that can see ten future trials with each requiring a choice between different reward options. In order to achieve the stated goal of OFT, the agent will now have to calculate the optimal choice path across all ten trials. There is only one method to obtain the exact optimal solution: the agent will have to consider all 2^10^ possibilities to determine the option-path that leads to the highest reward rate. In other words, on choice number 1, the agent will have to consider the effect of that choice on all possible future choices, and so forth. Clearly, such an exact solution is computationally intensive, with the combinatorial explosion making it infeasible for an animal to perform this computation beyond some limited number of trials.

Therefore, given that the exact solution is not attainable, how could one arrive at an approximate solution? Maximizing reward rate requires an animal to not waste time on a given trial, if that given trial presents options that are significantly worse than the environment as a whole. Hence, at its core, it requires the animal to be able to compare the reward rate available on a trial with the reward rate available on the session as a whole, so as to make an appropriate decision on that trial. A solution is to estimate the average reward rate of the ten known trials so as to estimate the reward rate achievable in the session as a whole. However, this is only possible under the assumption that the future is knowable. Therefore, let us now revoke this unrealistic assumption.

If the environment is assumed to be stationary (time-independent statistics), an approximate solution can yet be to use the past as a model to *predict* the immediate future, as the correlation between the immediate past and the immediate future is likely high. Of course this approximation would work only if the environment is stationary in time. Let us call such an epoch of time, modeled by the animal to be stationary, as a “session”. An example is a typical experimental session in the laboratory with unchanging reward statistics. Hence, a simple approximate solution to maximize reward rates over the session can be to maximize the reward rates over the time that one has spent in the session, i.e. instead of maximizing the reward rate prospectively in the session, maximize the reward rate achieved in the session *so far*. This solution contrasts with the solution presented by OFT shown in equation (5) because equation (5) assumed that the net reward rate for a given option is the reward rate achievable if one chose *only* that option. Instead, we use the past to compare the worth of a given option to the environment as a whole. This is the rationale for our theory, named Training-Integrated Maximized Estimation of Reinforcement Rate (TIMERR). In the remainder of this section, we formalize the above argument and show the implications it has on choice behavior.

#### 2.2.1 The past matters

**Figure 2.**
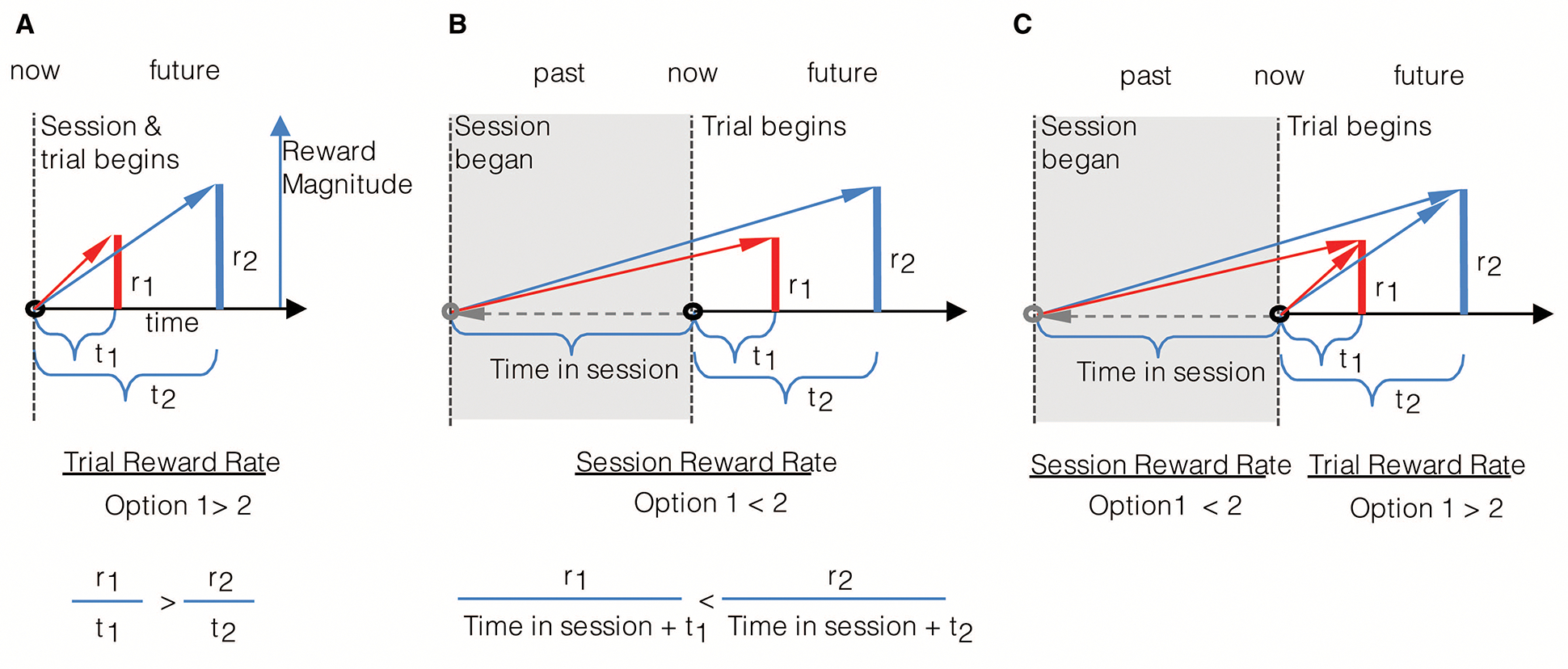
Does the past matter? A) Maximizing reward rate on a trial basis. An agent (black circle) presented with offers of reward that vary in magnitude and temporal displacement (($r_{1},t_{1}$) or $\left(r_{2},t_{2}\right)$) may decide upon a given option by calculating which offer yields the highest rate of reward in the trial. By normalizing reward magnitudes by the times to their future acquisition, Option 1 (red vector) in the instance given is found to have the highest trial reward rate. Trial reward rate is depicted graphically as the slope of the vector connecting the agent in the present moment (now) along the x-axis of time to the magnitude of future reward. B) Maximizing session reward rate. Alternatively, an agent presented with the same offers of reward as that in (A) may decide upon a given offer by calculating which option yields the highest session, rather than trial-rate of reward. Session reward rate is depicted graphically as the slope of the line connecting the agent on entry into the environment—its “past” self (grey circle) along the x-axis of time—to the magnitude of future reward. By normalizing the magnitude of a reward offered by the sum of the time already spent in the session plus the time to its future acquisition, Option 2 (blue vector) is found to have the highest session reward rate. C) Comparing choice behavior governed by trial reward rate versus session reward rate. By comparing the choices made by the agents in A and B to the same reward offers, it is apparent that decision-making governed by trial and session rate maximization are not equivalent, as they can lead to opposite choice behavior. Hence, if the objective is to gather the most reward while within a given environment, the past does in fact matter, as even evidenced when only considering elapsed time in the environment.

An agent, as in Figure 2, presented with offers of reward that vary in magnitude and temporal displacement ($\left(r_{1},t_{1}\right)$ or $\left(r_{2},t_{2}\right)$), may decide upon a given option by calculating which offer yields the highest rate of reward in the trial. By normalizing reward magnitudes by the times to their future acquisition, Option 1 (red bar) in this instance is found to have the highest trial reward rate. Trial reward rate is depicted graphically as the slope of the choice vector connecting the agent in the present moment (“now”) along the x-axis of time, to the magnitude of future reward (the slope of the red and blue vectors). Alternatively, an agent presented with the same offers of reward may decide upon a given offer by calculating which option yields the highest session, rather than trial rate of reward. Session reward rate is depicted graphically as the slope of the line connecting the agent on entry into the environment, its “past” self (grey circle) along the x-axis of time, to the magnitude of future reward. By normalizing the magnitude of a reward offered by the sum of the time already spent in the session *plus* the time to its future acquisition, Option 2 (blue bar) is found to have the highest session reward rate (slope of the blue vector). By comparing the choices made by the agents in A and B to the same reward offers, it is apparent that decision-making governed by trial and session rate maximization are not equivalent, as they can lead to opposite choice behavior. Hence, if the objective is to gather the most reward while within a given environment, the past does in fact matter, as even evidenced when only considering elapsed time in the environment.

Hence, what it means to select a reward option that “maximizes the rate of reward” depends, as evidenced in the prior figure, on the objective of the agent. An agent that makes its current choice based on maximizing the session reward rate—by considering how much time it has already spent in the environment—will outperform an agent that is wholly prospective (without including a model of the future based on the past) in its decision-making. Therefore, as a commonly stated goal within optimal foraging is to gather the most reward while within an environment, the former of the two agents would rightly be regarded as the rational of the two.

**Figure 3.**
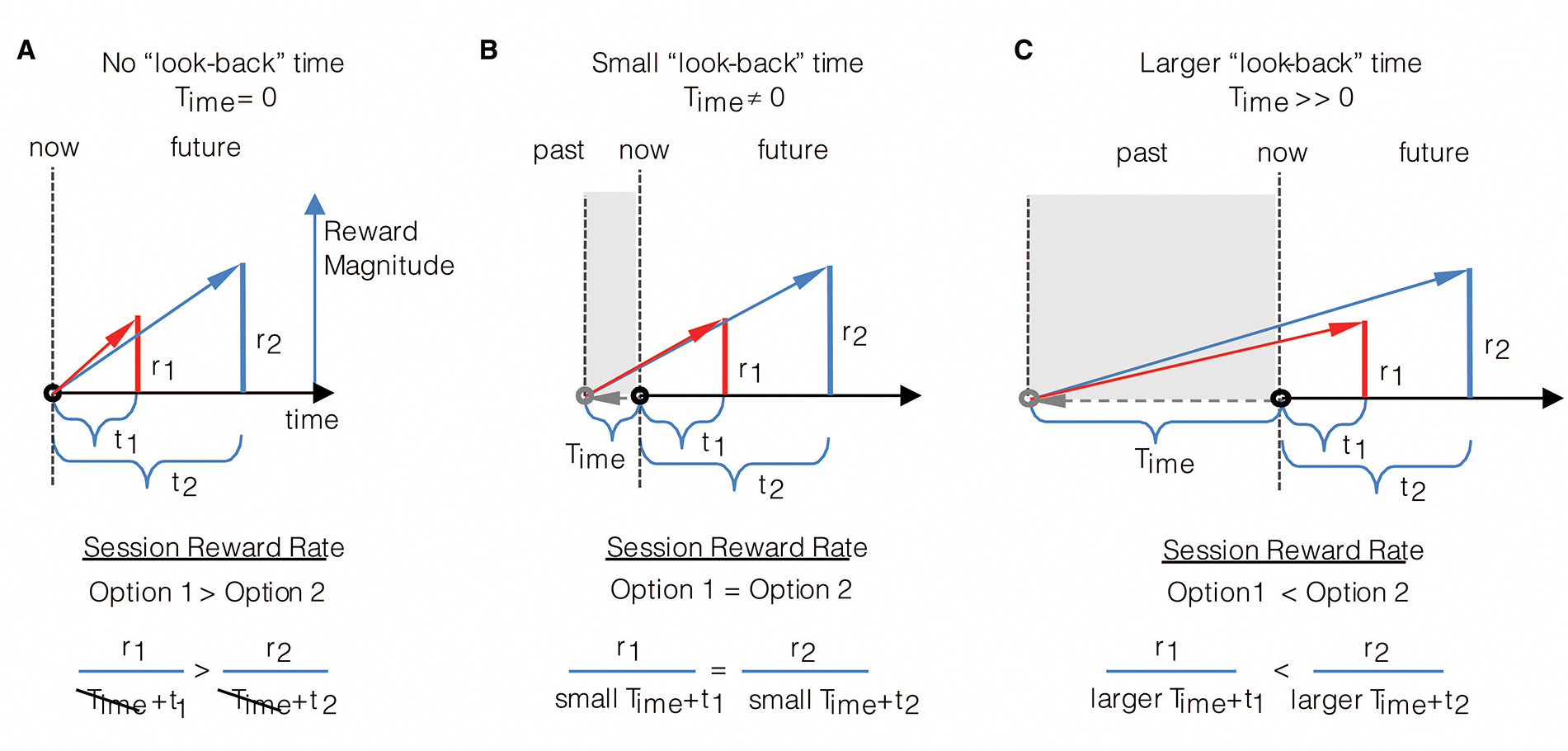
The effect of “looking-back” different amounts of time in evaluating realizable session reward rates. A) No “look-back” time. Should a session-reward-rate-maximizing agent look into its past no amount time (as potentially the case were it to have just entered into a foraging environment) it would choose Option 1 in the depicted example, as it yields the highest session reward rate. In this special case where $T_{\mathrm{ime}}=0$, evaluating reward options by maximizing session reward rate is equivalent to maximizing trial reward rate. B) Increasing $T_{\mathrm{ime}}$ (while decreasing the realizable session reward rates) does not affect the option chosen until $T_{\mathrm{ime}}$ reaches a value, as depicted in (B), wherein the reward options affect equivalent session reward rates. In this case, option 2 would be chosen as frequently as option 1. Growing the time in which the agent looks back into its past beyond this point results in a reversal of choice from Option 1 to Option 2, as depicted in (C). Therefore, the setting of $T_{\mathrm{ime}}$ critically affects the evaluation of delayed reward offers.

If wholly prospective decision-making is not characteristic of rational decision-making, is exhibiting a consistency in choice behavior to the same reward options (like in DUT)? Consider an agent (as depicted in Figure 3) that maximizes the session rate of reward. When being presented reward options upon entering an environment, as in panel A, the session-rate-maximizing agent selects option 1. However, if a quantity of time were to have passed in the environment prior to the presentation of the same reward options, as depicted in panel B, at that moment, choosing either option would be regarded as equivalent. Indeed, were even more time to have passed in the environment prior to presentation of the same options (as in panel C), option 2 would be selected. In all cases, the reward options presented are the same, making choice behavior appear to be inconsistent across the same reward offers. Only from the perspective of selecting the option that results in the highest session reward rate can choice behavior be regarded as actually being consistent, resulting in *different* reward options being selected.

### 2.3 TIMERR algorithm

Till now, we have considered the effect of the past on the valuation of reward offers under the special case that the agent has not acquired any (net) reward over that time. Of course, an agent may acquire reward in its past, and, should it have done so, the amount consumed need be taken into account in its current decision-making. Why is this so? In the prior figure, we provide an explanation as to why “looking back” in time would and should affect intertemporal decision-making even when the agent has not, to the present moment, accumulated any net reward. The benefit of looking back into one’s recent experience can be appreciated in another way, however, apart from simply the time spent in an environment, by considering, in addition, that a notion of experienced reward rate can be determined by normalizing the accumulated rewards harvested by the time spent harvesting in the environment. Therefore, one looks into the past not only to appreciate what interval reward rate should be maximized over, but to apprehend the rate of reward as already experienced in the environment.

Consider the case where the experienced environment has been a net positive one, meaning that the sum of acquired rewards (denoted by R) over the “look-back” time ($T_{\mathrm{ime}}$) has a positive value. Whatever the rate of reward experienced over this past look-back interval, offers of future reward must yield a rate greater than the experienced reward rate to ensure that the reward rate of the session increases. Therefore, looking back serves the purpose of determining the rate of reward that one should expect of an environment: reward options that decrease the experienced rate of return signify that they are subpar options that should not be taken.

**Figure 4.**
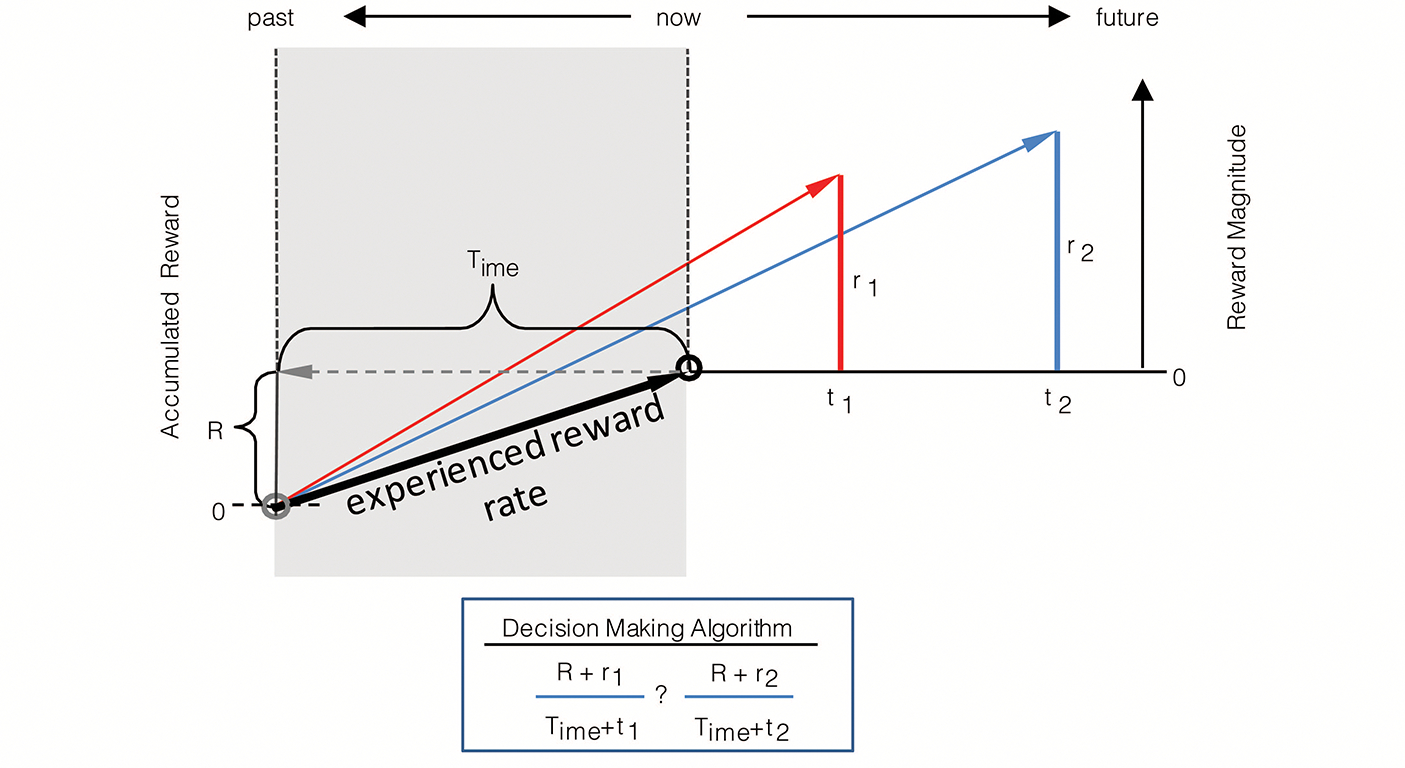
The TIMERR decision-making algorithm and its graphical depiction. The TIMERR algorithm can be understood simply as choosing the option that leads to the highest reward rate over the experienced interval within an environment up to and including the time to the future reward option. Reward accumulated within the environment (R) over the time the agent looks back into its past $\left(T_{\mathrm{ime}}\right)$ yields the experienced reward rate ($R/T_{\mathrm{ime}}$, slope of the black vector) which is added to the reward rate of a future option $\left(r_{i},t_{i}\right)$ to yield a realizable reward rate having chosen that option. The agent then selects the realizable reward rate with the highest rate of return. A caveat is that the agent may forgo an option even if it evaluates to the highest realizable reward rate should that rate be less than the experienced reward rate (see section 2.2.5). Black circle: the agent at the present moment of time, “now”. Grey circle: the agent’s past self on entry into an environment at which time it has yet to accumulate any reward within it. Slope of the red and blue vectors: the realizable reward rates of the reward options $\left(\left(r_{1}t_{1}\right)\left(r_{2},t_{2}\right)\right)$. Left-hand y-axis: the amount of accumulated reward in the environment from entry into the environment until the present moment, “now”. Backward-pointing grey vector: the magnitude of the grey vector indicates the time over which the agent looks into its past. Its termination points to the amount of reward so far accumulated over that interval of time. Right-hand y-axis: the magnitude of future reward options.

The rate of experienced reward can be incorporated into the graphical depiction as previously given for the (special) case—wherein the rewards accumulated in the environment sum to zero, as in Figure 3—to yield a depiction of the decision-making algorithm in the general case where accumulated reward can take on any value, be it positive or negative (Figure 4). As in Figure 3, the right hand y-axis in Figure 4 plots the magnitude of future reward offers. Also as in Figure 3 (though there not labeled as such), the left hand y-axis plots the magnitude of accumulated past reward, the value of which the backwardly pointing grey vector terminates. Whereas in Figure 3, the backward pointing grey vector points to a value of accumulated past reward equal to zero, in Figure 4 it points to some positive value of accumulated past reward, R, thereby shifting the origin of the left-hand y-axis representing accumulated past reward downward. The rate of experienced reward ($R/T_{\mathrm{ime}}$) is thus graphically depicted as the slope of the line connecting the agent’s past self with its current self (black vector). By depicting accumulated past rewards and future reward offers in this manner, the relationship between the past reward rate and the offered future reward rates can be seen to sum to yield the realizable session reward rates (the slope of the red and blue vectors). Given known reward offers, deciding between offers is then simply a matter of determining which opportunity yields the highest session reward rate, and, should it exceed the experienced reward rate, choosing that reward option. Expressed another way, offered reward rates are added to the experienced reward rates to determine session reward rates, the largest of which (so long as it exceeds that which is already experienced) is then selected. This can be expressed as the following choice algorithm:

(7) $$\mathrm{Choose}\;max\left(\frac{r\;+\;R}{t\;+\;T_{\mathrm{ime}}}\right)$$

#### 2.3.1 Expressing the TIMERR algorithm in terms of subjective value

**Figure 5.**
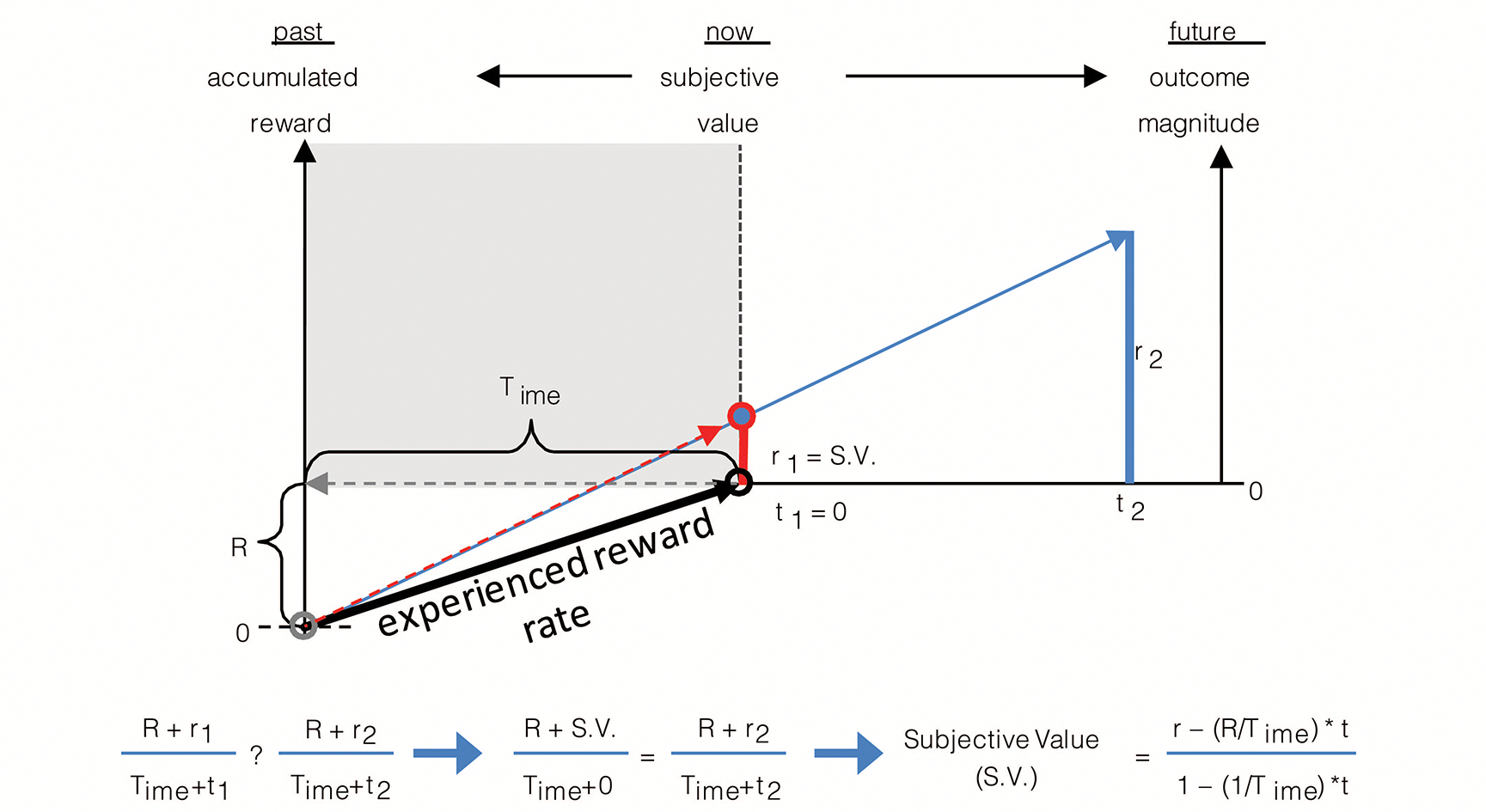
Subjective value derived from the TIMERR algorithm and graphically depicted. Under the TIMERR conception, a future offer of reward of known magnitude and temporal displacement $\left(r_{2},t_{2}\right)$ is equivalent to an offer of reward presented at the current moment of time $\left(r_{1},t_{1}\right)$ that effectuates the same realizable session reward rates (the slope of blue and red choice vectors), as graphically depicted above. This graphical depiction of TIMERR, thus provides a ready visual means of apprehending the subjective value of any outcome; it is the y-axis intercept at the present moment of time (red circle with blue fill represents the subjective value of option1 being equivalent to the subjective value of option 2). Therefore, as subjective value is the magnitude of reward given now that is perceived as being equivalent to a larger later reward, the TIMERR algorithm can be used to derive an expression for subjective value by setting the future reward option $\left(r_{2},t_{2}\right)$ as equal to a present reward option $\left(r_{1},t_{1}\right)$ where $t_{1}=0$) and solving for $r_{1}$, the subjective value of the larger later offer. The expression for subjective value, so derived, is given in equation (8). Note that in this instance, reward options result in realizable session rates of reward that exceed the experienced reward rate (slope of the black vector) of the agent (black circle), and are accordingly positive subjective values. Were an option to result in a realizable session rate below the experienced rate of reward, its y-axis intercept would be negative, resulting in a negative subjective value.

Subjective value is the magnitude of reward available “now” (see Figure 5) that is perceived as being equivalent to a larger later reward. Indeed, decision-making using the TIMERR algorithm, rather than being a determination of session reward rate as in Equation (7), can alternatively be re-conceptualized as selecting the option offering the highest positive subjective value. In this reconceptualization of the TIMERR algorithm, the subjective value of a future reward option is equivalent to the magnitude of reward given “now” that yields the same session reward rate. Therefore, the graphical depiction of the TIMERR algorithm in the preceding figures provides a ready means of determining the subjective value of any presented offer: subjective value is the y-intercept of the offer’s session rate vector at the present moment of time.

Consider the options presented in Figure 5. Option 2 is a reward of magnitude, $r_{2}$, set to occur at a delay, $t_{2}$, in the future. Selecting this option will yield a session reward rate given by the slope of the blue vector. Option 1 is a reward of magnitude, $r_{1}$, occurring now (t=o) that yields the same session reward rate as Option 2 (the slopes of the red and blue vectors are equal). As subjective value is the magnitude of reward given now that is regarded as equivalent to a larger later reward, setting $t_{1}$ to zero and solving for $r_{1}$ *is* solving for the subjective value of Option 2. Therefore, by so expressing reward options as an equality between a future offer and an offer presented now that yields the same session reward rate, the decision-making algorithm of TIMERR can be used to derive the subjective value of any reward. As shown in the figure, this can be expressed as

(8) $$SV(r,t)=\frac{r\;-\;a_{est}t}{1\;+\;\frac{t}{T_{ime}}}$$

where $a_{\mathrm{est}}=R/T_{\mathrm{ime}}$ is the past reward rate, and SV(r,t) is the subjective value of a reward of magnitude r delayed by t.

The derivation of subjective value from the TIMERR algorithm (Figure 5, Equation (8)) provides an opportunity to appreciate this deceptively simple process from a different perspective. The subjective value of a future offer is defined, in part, by the numerator, being the magnitude of the offered reward, r, less the amount of reward expected to occur in lieu of taking the offer. The amount of reward expected to occur in lieu of taking the offer is the opportunity cost, ($a_{\mathrm{est}}\;t$), which is determined by multiplying the experienced rate of reward ($a_{\mathrm{est}}=R/T_{\mathrm{ime}}$) by the time required for the offered reward’s acquisition (t). The denominator is the explicit cost of time itself, and is notable in that the general form it takes bears resemblance to that of typical discounting functions, save for the fact that they have a free-fit parameter governing the steepness of discounting in time. Here, the discounting constant (like k in Equation (2)) for time is not a free-fitting parameter of uncertain biological meaning but rather is the reciprocal of the look-back time, $T_{\mathrm{ime}}$. By so expressing TIMERR in terms of subjective value, the experienced reward rate is understood as governing opportunity cost, whereas $T_{\mathrm{ime}}$ controls the steepness of temporal discounting.

#### 2.3.2 The effect of changing the look-back time, Time, and the magnitude of accumulated reward, r, on the valuation of given reward options.

**Figure 6.**
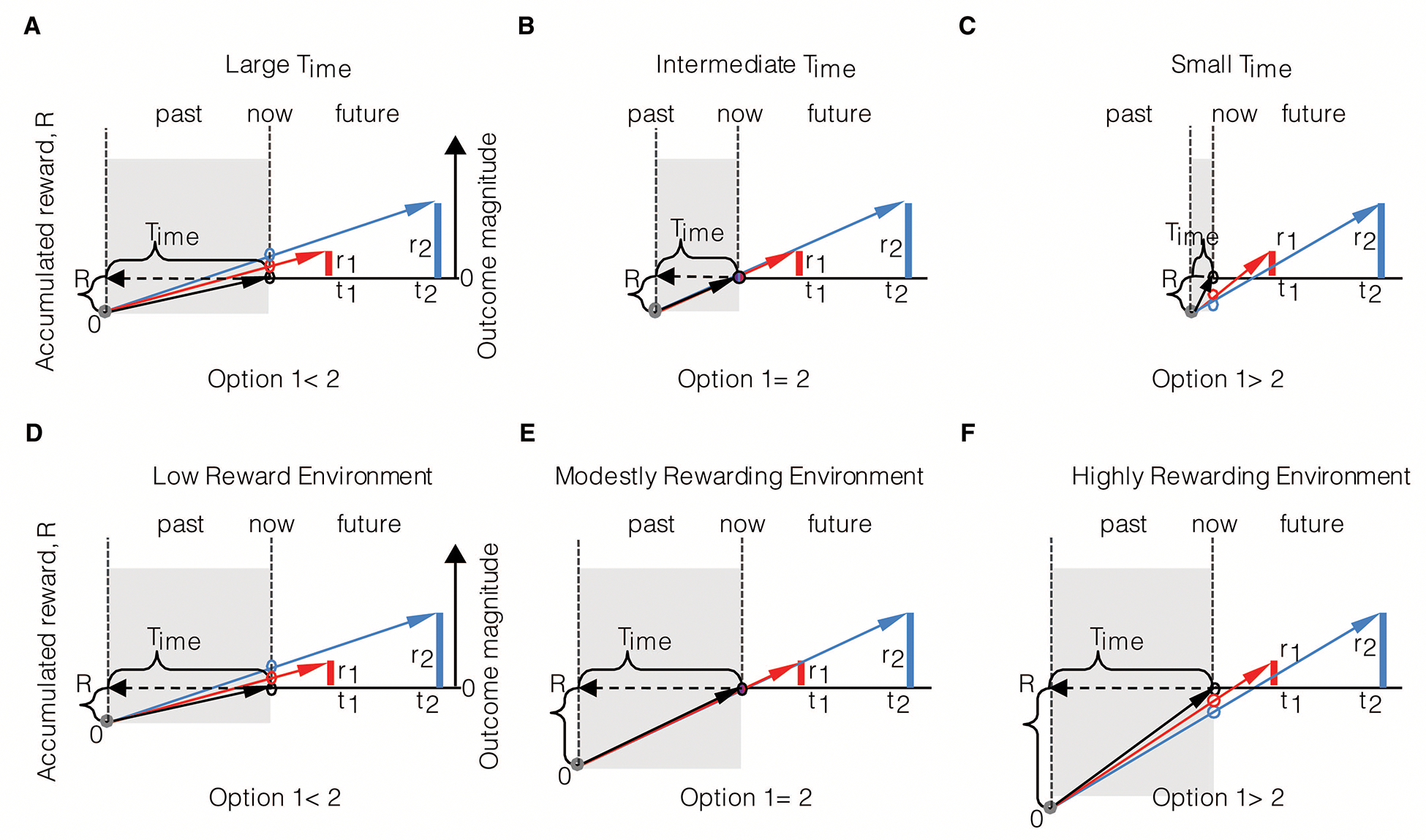
The effect of changing the look-back time, $T_{\mathrm{ime}}$, and the magnitude of accumulated reward, R, on the valuation of given reward options. (A-C) The effect of changing $T_{\mathrm{ime}}$ on the valuation of a pair of known reward options $\left(\left(r_{1},t_{1}\right),\left(r_{2},t_{2}\right)\right)$ evaluates to different session rates of reward, and therefore, different choices, such that the agent in (A) that looks back a relatively longer amount of time selects option 2, whereas the agent in (B), looking back an intermediate amount of time regards the options as equivalent, and finally, where the agent in (C), that looks back a brief amount of time, selects option 1. (D-F) The effect of varying the experienced reward rate ($R=a_{\mathrm{est}}t$) obtained from a low (D), modest (E), or high (F) reward environment, on the valuation of the same reward options. D) When having experienced a low rate of reward, the offers presented evaluate such that the larger later reward option is greatest. E) In a more rewarding environment, both offers evaluate to the same subjective value, being zero, as neither advances nor retards the reward rate experienced. F) In an even more rewarding environment, the same reward options evaluate such that the larger later reward yields the greater of the two subjective values. Parametrically changing the look-back time, $T_{\mathrm{ime}}$, or the magnitude of accumulated reward, R, is thus shown to affect the experienced reward rate and therefore, the valuation of future reward options, thereby determining option selection.

What then is the effect of different look-back times and magnitudes of accumulated reward on the subjective value of given reward options? Let us first consider the effect of the look-back time, $T_{\mathrm{ime}}$, as we have in prior figures (Figures 2 and 3), but from the perspective of determining the subjective value of rewarding options. An agent, as in Figure 6, that looks-back a relatively large amount of time into its past to calculate its experienced rate of return in the environment regards the larger later reward option as the option with the highest subjective value. However, if the agent’s look-back time were to be less extensive, an intermediate value of $T_{\mathrm{ime}}$ exists that results in the agent regarding the same offers as subjectively equivalent. In the particular instance given in Figure 6B, the subjective values of option 1 and 2 are equivalent and have a value of zero, for choosing either option would neither advance nor retard the reward rate experienced in the environment. If $T_{\mathrm{ime}}$ were to be smaller still, the smaller earlier option would be evaluated as having the greater subjective value (Figure 6C). Therefore, the amount in which the animal looks back into its past affects choice behavior to what otherwise would be regarded as the same reward options, as mentioned previously.

How does the amount of accumulated reward acquired by the agent over a fixed amount of time affect the evaluation of subjective value? Consider the agent with a fixed look-back time ($T_{\mathrm{ime}}$) that is offered the same rewarding options in three different environments yielding low, modest, and high (Figure 6D–F) experienced reward rates. Having experienced a low rate of reward (Figure 6D), the offers presented are evaluated such that larger later reward option has the highest subjective value. In the modestly rewarding environment (Figure 6E), both offers are equivalent and have a value of zero, as choosing either option, again, neither advances nor retards the reward rate experienced. Finally, in the higher reward environment (Figure 6F), the same reward options presented to the animal evaluate such that the smaller earlier option has the greater subjective value. Therefore, as with $T_{\mathrm{ime}}$, the amount of reward accumulated $\left(R=a_{\mathrm{est}}t\right)$ over that look-back time affects the evaluation of subjective value to the same rewarding options. The significance of these effects is that ostensibly inconsistent choice behavior may manifest from the consistent application of the TIMERR algorithm maximizing reward acquisition.

### 2.4 When should an offered reward be forgone?

**Figure 7.**
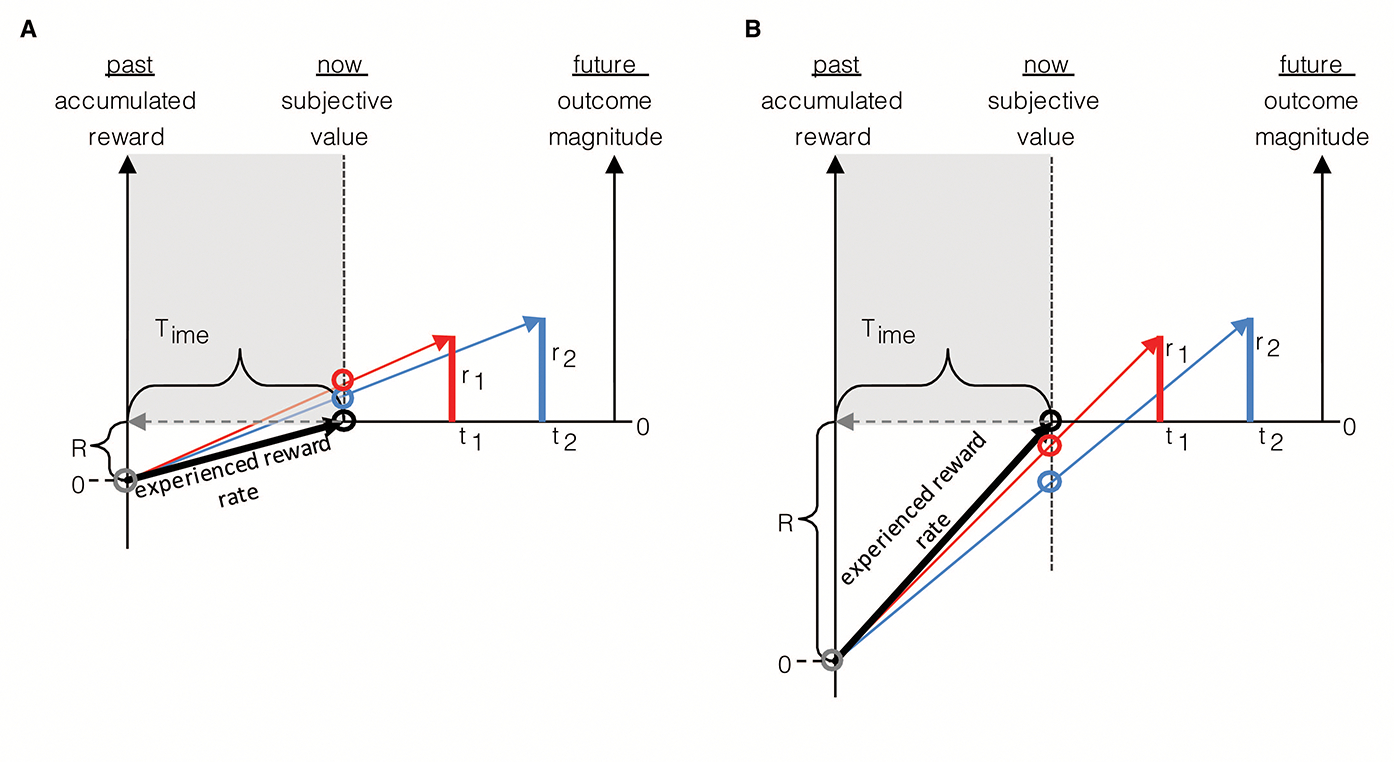
When should an offered reward be forgone? A) An agent, having experienced a net positive rewarding environment, is presented with reward options 1&2 and selects option 1 as it yields the highest session reward rate, or equivalently, the highest subjective value (red circle). B) The same reward options presented again to the agent, but subsequent to having obtained a higher experienced rate than that in (A), are now forgone, as their respective realizable session reward rates are less than the experienced reward rate, and thereby evaluate to negative subjective value. So, despite the fact that the offer’s session reward rates are positive, the agent forgoes the reward options presented in the instance presented, as its experience in the environment indicates that a superior reward option is expected to occur in the future. The environment shown here is similar to that shown in Figure 6F. Hence, even in Figure 6F, if the agent had an option to choose either reward or forgo both, both would be forgone.

When should reward options be foregone? TIMERR dictates that the option that yields the highest *positive* subjective value is chosen, so ensuring that the most reward possible is garnered while in the environment till now. Consider, for instance, the agent depicted in Figure 7A, which, having experienced a net positive rewarding environment, is presented with reward options 1&2. In this case, the agent selects option 1, in that that option yields the highest achievable session reward rate, or equivalently, the highest subjective value (red circle). Should the agent have experienced an even greater reward rate (slope of black vector) after continuing to forage in the environment, as in Figure 7B, the same offers now evaluate to negative subjective values, as their session reward rate vectors now have negative y-axis intercepts. So, despite the fact that the offer’s session reward rates are positive, the agent should and would (if given the option) forgo the reward options presented in this instance, as they yield session rates of reward that are less than the experienced reward rate.

#### 2.4.1 Choosing a punishment over a reward?

**Figure 8.**
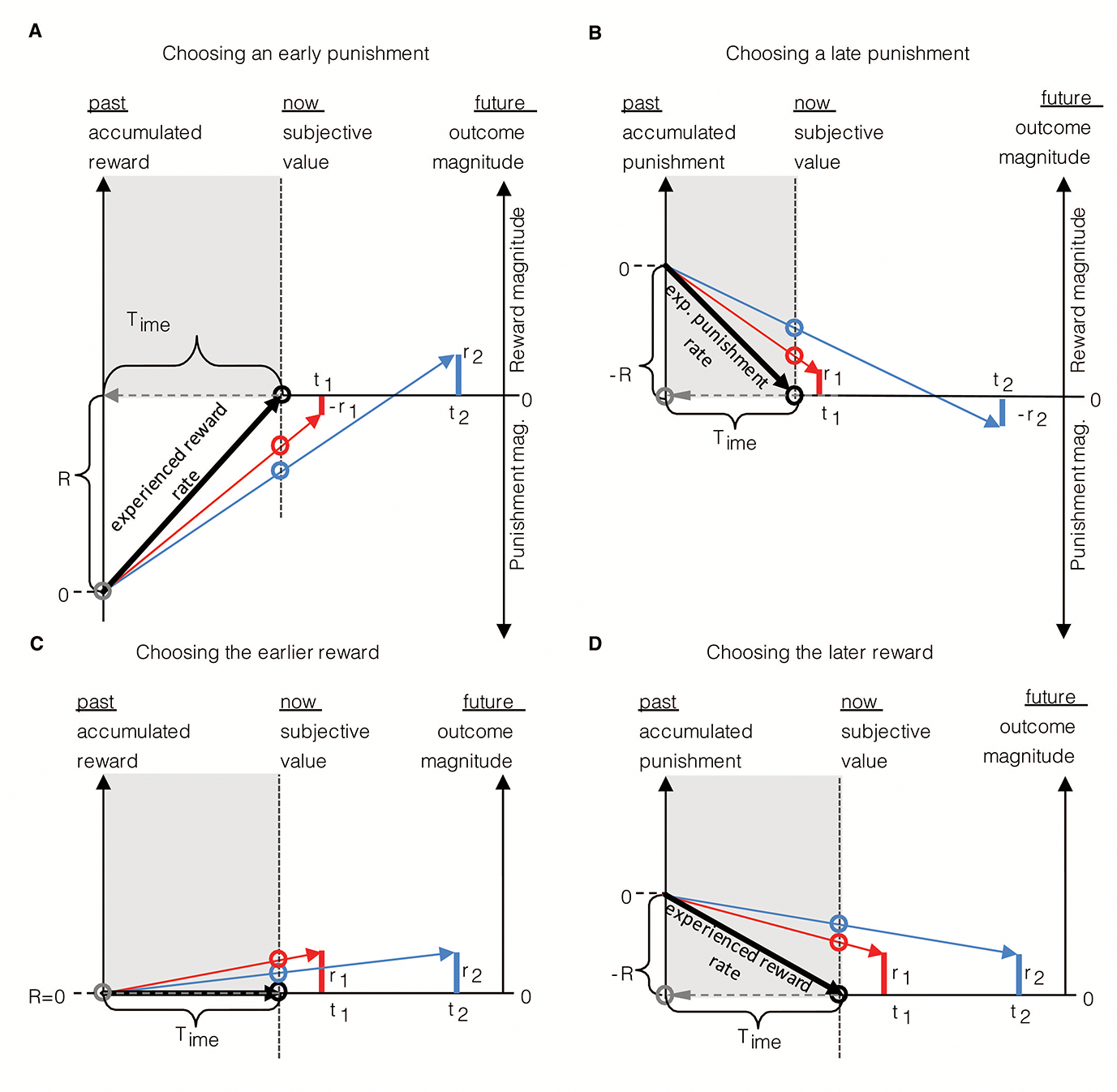
Choosing a punishment over a reward? A) Given a net rewarding environment, outcome options between an early punishment and a later reward may be presented to an agent such that evaluation of the outcomes yields a higher subjective value for the early punishment. In this case, note that the subjective values of either offer are negative, and therefore would be forgone by the agent. However, if continuation of foraging for reward is contingent on making a choice between the options at hand (as is commonly the case in forced-choice experiments), the agent would select the early punishment as it incurs the least cost to the animal. B) Conversely, an agent may choose a later punishment over reward were it to have been experiencing a net negative reward (punishing) environment. In the case depicted, the later punishment is selected as it has the highest subjective value. C&D) When might an agent prefer a later reward of a given magnitude over an earlier reward of the same magnitude? C&D depict the case where an agent selects the earlier reward in one case (C; a net neutral environment) and the latter reward in another (D; a net negative rewarding environment).

Presently, we have only considered intertemporal decision-making with respect to rewarding outcomes. Of course, behaviorally significant events can be not only rewarding but punishing as well. Faced with an option to choose a punishing or a rewarding outcome, would an agent ever choose the punishing one? The agent in Figure 8A is presented such an option between a punishment at a short delay ($r_{1},t_{1}$) and a reward at a long delay ($r_{2},t_{2}$). If it has experienced a net positive reward environment as depicted, both offers in this instance evaluate to negative subjective values (as their realizable session reward rates are lower than that of the experienced rate of reward). Therefore, in keeping with the prior section, the agent would forgo both options. However, should the possibility of additional trials be contingent on completing the current one, as is often the case experimentally in forced-trial designs, selecting the early punishment over the later reward would be optimal as it incurs the least cost. In this case, then, an early punishment would be chosen over a later reward.

Might then a later punishment ever be selected over an earlier reward? Envision an environment where the net accumulated reward is negative (i.e., there is an experienced rate of punishment). As depicted in Figure 8B, an agent in such an environment, given the options depicted between an early reward and a later punishment, would pick the later punishment. The agent would pick the later punishment because it effectuates the greatest positive change (greatest decrease in the rate of punishment) in the session reward rate, thereby expressing the highest positive subjective value.

The examples in Figure 8A&B give insight into what circumstances lead to the unintuitive selection of earlier or later punishment over a reward. What, however, is the effect of changing the environment to a net negative one on the valuation of given offered rewards, as in Figure 8C&D? In Figure 8C the agent is in a net neutral environment and is given an option between two rewards that are of the same magnitude, but displaced in time. Here, the subjective value of the early option is the greatest, as the added cost of time diminishes the subjective value of the equally sized, later reward. However, presented with the same reward options but now when having experienced a net negative rewarding environment, the same agent selects the later over the earlier reward. Why? In this case, it is not only the magnitude of reward that decreases the rate of punishment, but also its associated delay; time, rather than having an associated cost, can under some circumstances have an associated *gain*.

These instances, albeit unusual, are instructive in that no trial-reward-rate-maximizing agent would ever choose to forgo a reward option (as in section 2.2.5), nor select the punishment option (as in this section), nor pick a later instance of an equivalently sized reward.

#### 2.4.2 Re-expressing subjective value as a discounting function, and the effect of $T_{\mathrm{ime}}$.

**Figure 9.**
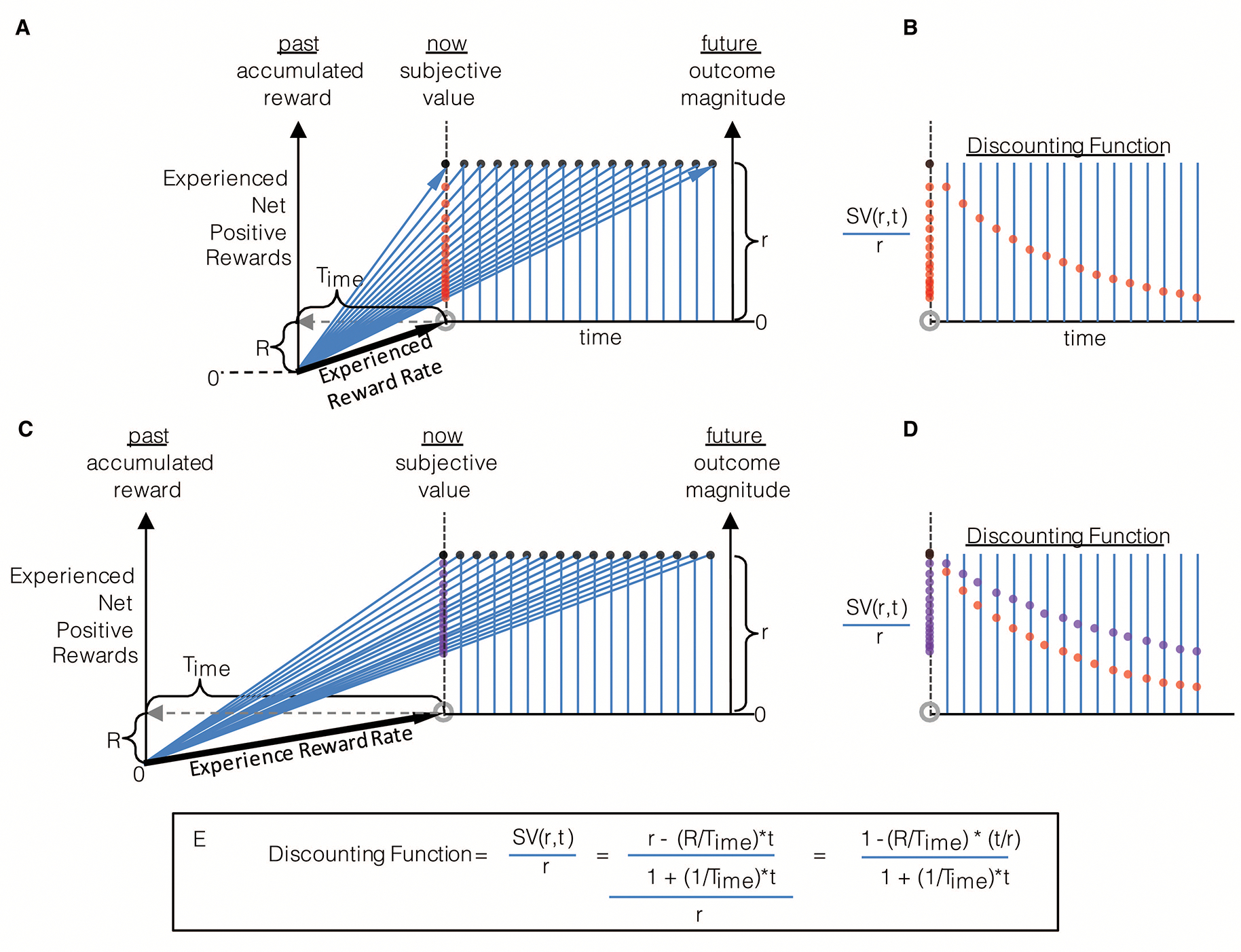
Subjective value expressed as a discounting function, and, the effect of $T_{\mathrm{ime}}$. A. The realizable reward rate (the slope of the blue vectors) of an offer of magnitude, r, starting from the present moment and arrayed at fixed intervals into the future (black dots), is depicted for an agent that has accumulated a total reward R, over its look-back time, $T_{\mathrm{ime}}$. Red dots along the “now” y-axis indicate the subjective value of the offer at corresponding delays (the magnitude of the offer needed now that would be treated as subjectively equivalent to the later larger reward). Note that as the offer recedes in time, the realizable reward rate, and therefore subjective value, correspondingly drops, but diminishingly so. B. Expressing the subjective values derived in (A) in terms of a discounting function. By replotting the subjective values of the reward option, r, across fixed intervals of t, to their corresponding delays, the drop in subjective value of a given offer with time can be appreciated. Note, here, that the y-axis, equivalent to that shown in (A), is now re-expressed as subjective value per unit of offered reward. The resulting temporal discounting function is hyperbolic in shape, according with the preponderance of experimental observation. C. The effect of looking back more distantly into the past. For the same offers of reward as that considered in (A), an agent looking back more distantly into its past (but experiencing the same accumulated reward) will evaluate those offers as having higher realizable rates of return and therefore correspondingly higher subjective value (purple dots along the y-axis, “now”). Replotting those subjective values as in (B), one then observes that agents that look back over greater stretches of time into their past, treat nominally the same reward offers as being more valuable, and therefore generate apparent discounting functions that are less steep. In short, the larger the value of $T_{\mathrm{ime}}$, the less steep apparent discounting; alternatively, the more patient the agent. E) Discounting Function. Discounting functions convey subjective value (SV(r,t)) of an offer as a function of time, expressed as a fraction of the offer’s outcome magnitude. Replacing SV(r,t) for the right-hand side of equation (8) and then simplifying yields the TIMERR algorithm expressed as a temporal discounting function (Equation (9)). Of central importance is that the term controlling the steepness of temporal discounting function is not a free-fit parameter of uncertain biological meaning but rather is the reciprocal of the look back time of the animal $\left(1/T_{\mathrm{ime}}\right)$.

The degree to which one looks back into the their past as well as the magnitude of reward accumulated over that time have both been shown to fundamentally affect the valuation of future rewards; yet, we have not systematically examined the effect of delaying a given outcome on its valuation. Consider the agent in Figure 9A that has experienced a net positive reward, R, over its look-back time, $T_{\mathrm{ime}}$. If presented a reward of magnitude (r) at the present moment of time, how does this reward’s temporal displacement into the future affect its valuation? From the perspective of its realizable reward rate in the environment, this rate (the slope of the blue vectors) decreases as the reward is displaced further into the future. Equivalently, for each delay examined, the subjective value can be calculated (Equation (8), see - Figure 5) and appreciated graphically as the corresponding vector’s y-intercept at the present moment of time (“now”). As a given sized reward recedes into the future, its subjective value correspondingly drops with a decreasing rate. Indeed, by re-plotting the subjective values to their respective delays, as in Figure 9B, the manner by which subjective value decreases with time can more readily be appreciated; it is in fact—when expressed as a fraction of the actual outcome magnitude—the “temporal discounting function”. The discounting function is thus expressed mathematically as

(9) $$D(r,t)=\frac{1\;-\;\frac{a_{est}t}{r}}{1\;+\;\frac{t}{T_{ime}}}$$

By so re-expressing subjective value, evaluation of an offer by the TIMERR algorithm can be understood in terms of an *apparent* discount function. One need note, however, that the drop with time of 1) the slope of realizable reward rate, 2) the subjective value, and 3) the discounting function are all mathematically equivalent means of understanding the TIMERR algorithm. So, while expressing intertemporal decision-making by TIMERR in terms of a discounting function is of use in relating it to established notions of discounting functions, from the perspective of the TIMERR algorithm, there is no requirement to actually *have* a discounting function. As such, its observation is but a consequence of the TIMERR algorithm rather than evidence of an entity (with agency) that is applied to offers so as to determine their subjective value, as commonly held. Nonetheless, the shape of the apparent discounting function that derives from the TIMERR algorithm is hyperbolic in form, according well with the preponderance of experimental observation (e.g., Ainslie, 1975; Ainslie & Monterosso, 2003; Kalenscher & Pennartz, 2008; Loewenstein & Prelec, 1992).

An important feature of TIMERR then, is that the *apparent* discount function is controlled by the amount of time that the agent looks back into its past, $T_{\mathrm{ime}}$. The effect of looking back in time on the steepness of discounting is readily apparent by comparing Figure 9A&B to panels C&D. The longer the agent looks back, the more patient the agent appears to be, being more willing to wait for the same magnitude of reward at a greater temporal delay. Conversely, the less the agent looks back into its past, the more impulsive, seemingly, the agent. Therefore, whereas extant models of temporal discounting inject a free-fit parameter of unknown biological meaning so as to best approximate experimental observation, the feature that wholly controls the steepness of discounting in the TIMERR conception is the degree to which the agent looks back into its past.

#### 2.4.3 The Magnitude Effect

The applicability of the TIMERR conception to understanding intertemporal decision-making rests on making an accounting for hallmark observations well established in the behavioral literature. Above, we have ascertained that the appearance of hyperbolic discounting would be observed in an agent that operates in a manner consistent with the TIMERR algorithm. Since the TIMERR algorithm maximizes reward under experiential constraints, temporal discounting that exhibits a hyperbolic form resulting from TIMERR is not irrational, but rather reward maximizing. Might other so-called “anomalous observations” (in economics, see Frederick et al. (2002), Kalenscher and Pennartz (2008), and Loewenstein and Prelec (1992) for reviews) in intertemporal-decision making be similarly predicted by the TIMERR algorithm, and if so, be shown not to be deficiencies in, but rather signs of, rational decision-making?

**Figure 10.**
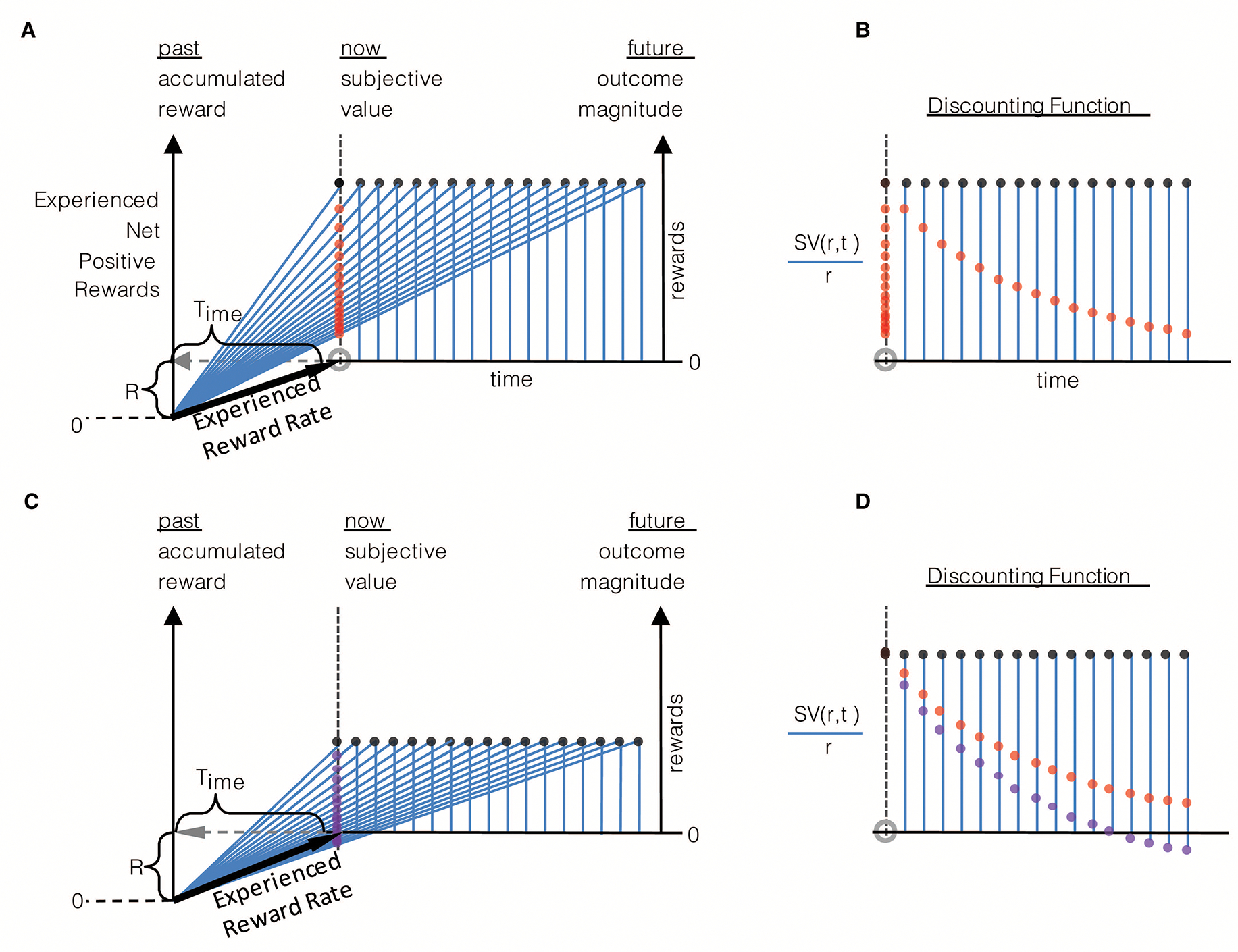
The Magnitude Effect is a consequence of experientially constrained reward rate maximization as conceptualized by TIMERR. A) Realizable reward rates (the slope of blue vectors) to a reward of a given size but arrayed in time illustrates how the rate of return decreases, diminishingly, as the reward recedes in time. The subjective value of each offer can be found as its y-intercept at the present moment (“now”). B) Discounting function of the temporally arrayed offer in (A). By replotting each subjective value to its corresponding temporal interval, subjective value is observed to decrease hyperbolically with temporal delay. C) Realizable reward rates are again plotted but to a reward arrayed through time of half the magnitude as that in (A). The subjective value of each offer is again found as its corresponding y-intercept at the present moment (“now”). D) The discounting function of the temporally arrayed offer in (C, purple) compared to that derived in (B, red) demonstrates that the steepness of discounting is less steep for the larger of the two rewards, i.e., the “Magnitude Effect”. Subjective value is normalized to the reward magnitude when plotting the discounting function.

One such hallmark observation is that of the “magnitude effect”, wherein the steepness of observed temporal discounting is dependent on the magnitude of the offered reward, such that larger rewards exhibit shallower discounting than smaller rewards. Might the magnitude effect be a natural consequence of the TIMERR algorithm? As in the prior figure, subjective value can be determined for a reward of a given magnitude that is arrayed across temporal delays (Figure 10A), and re-plotted as a temporal discounting function (Figure 10B). Consider, now, the subjective values for a reward half that magnitude arrayed across the same range of delays (Figure 10C). By similarly re-plotting subjective values, normalized to the magnitude of the offer as before, it is apparent that the steepness of discounting decreases the larger the offered reward magnitude (Figure 10D). Therefore reward rate maximization while in an environment results in the appearance of a discounting function that is sensitive to the magnitude of offered reward.

#### 2.4.4 The Sign Effect

**Figure 11.**
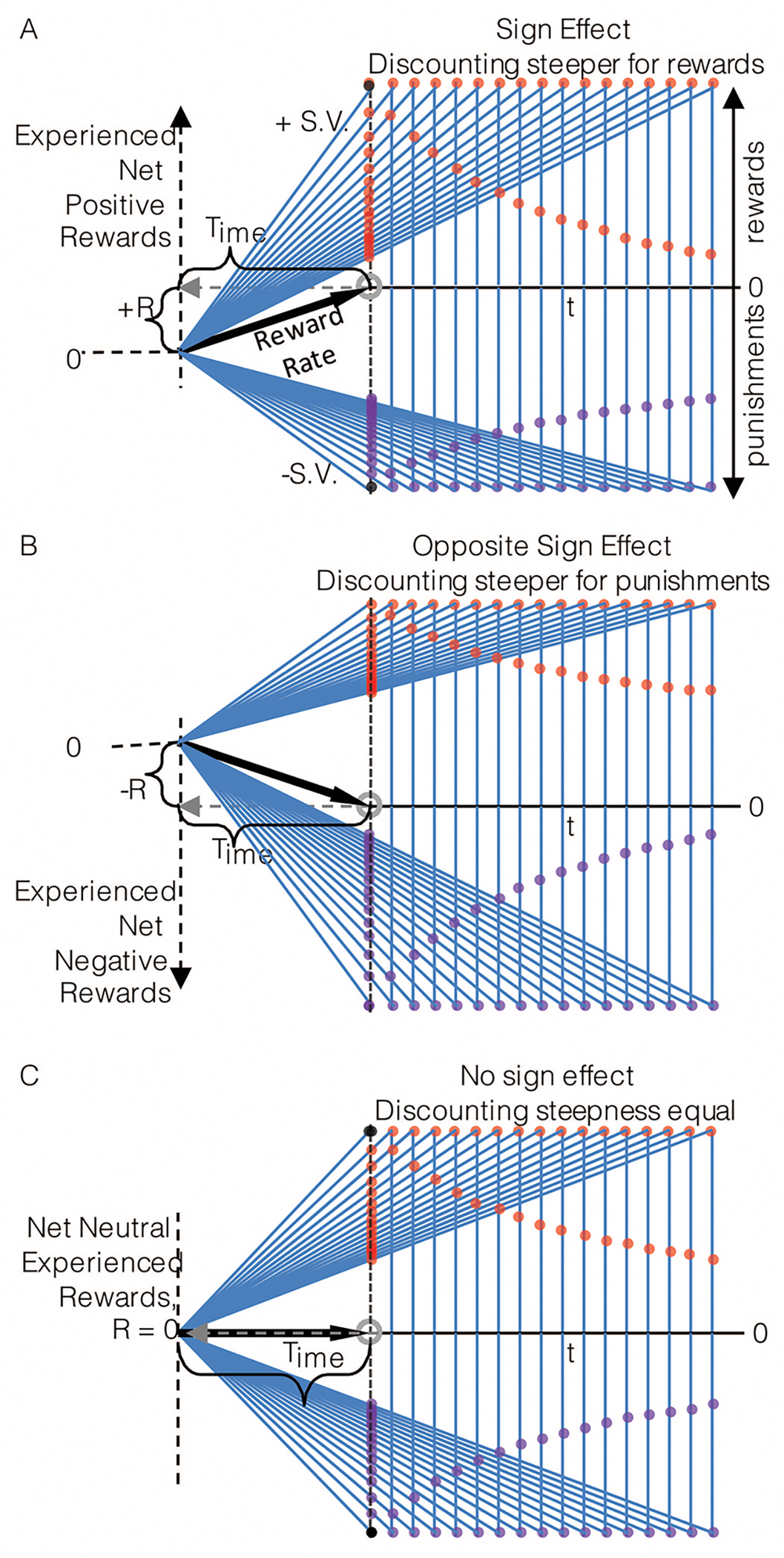
The “Sign Effect” as explained by the TIMERR conception, in net positive, negative, and neutral reward environments. A. The sign-effect in a net positive reward environment. Experienced reward history generates a bias in evaluating equivalently sized outcomes of opposite sign, leading to an apparent discounting function for rewards that is less steep than that for punishments. B. The “sign-effect” as predicted by TIMERR for an agent having experienced a net negative reward environment also exhibits asymmetric discounting functions for rewards and punishments, yet here, punishments rather than rewards discount less steeply, i.e., the sign of the sign-effect is reversed. C. The absence of a net positive or negative reward experience leads to the absence of bias in evaluating the worth of rewards and punishments. Under this condition, outcomes of equivalent magnitudes, be they rewards or punishment, discount at the same rate. Therefore, given a net neutral reward experience within an environment, the sign effect will not be observed.

Another so-called anomalous behavior observed in intertemporal decision-making is that of the “sign-effect” wherein outcomes of equal magnitude but opposite sign are observed to discount at different rates; specifically, that rewards discount more steeply than punishments. The origins and necessary conditions of this effect are evident in the graphical depiction of TIMERR in Figure 11A–C. Here, an agent considers rewards and punishments of equal magnitude arrayed into the future under three different environments: net positive (Figure 11A), net negative (Figure 11B), and net neutral (Figure 11C) outcome environments. Within the first environment (Figure 11A), the agent has experienced a net positive accumulation of reward, R, over its look back time, $T_{\mathrm{ime}}$, and therefore has a positive experienced rate of reward (black vector). Following prior convention, realizable reward rates given any outcome selection are depicted as the slope of the choice vectors (blue) connecting the agent’s past self to its future self having chosen an option. The intersection of these choice vectors with the y-axis at the present moment of time yields the outcomes’ respective subjective values. Re-plotting these subjective values to their corresponding temporal delays produces the temporal discounting functions for the positive and negative rewards (as all outcomes considered are of unit magnitude, the y-axis of subjective value is equivalent to a y-axis of subjective value normalized by unit outcome magnitude). The origin of the sign-effect in a net rewarding environment is then understood as the effect of reward magnitudes countermanding opportunity cost, while punishment magnitudes exacerbating opportunity cost.

In Figure 11A we note that the sign-effect arises due to the opposite impact of magnitude of rewards and punishments on opportunity cost. Suppose, however, that an agent has experienced a net negative (punishing) environment (Figure 11B). Should the sign-effect be observed? In that outcomes of equal magnitude but opposite sign are observed to discount at different rates, yes, a sign-effect would, under the TIMERR conception, be observed. However, should the “sign-effect” be defined as rewards discounting more steeply than equally sized punishment, then no. Rather, TIMERR predicts that in net negative environments it is *punishments* that discount more steeply than equally sized rewards. The sign of the “sign-effect” flips in net negative environments. Why? Whereas in net rewarding environments there is an opportunity cost associated with any reward in time, in net punishing environments there is an opportunity gain associated with any punishment in time. Therefore, the origination of this sign-effect in a net punishing environment is understood as the effect of punishment magnitudes countermanding opportunity gain, whereas reward magnitudes combine with opportunity gain.

What then of the sign-effect if the agent has experienced a net neutral reward environment? In this case (Figure 11C) there is no opportunity cost/gain associated with any reward or punishment. Therefore, rewards and punishments of equal magnitude neither countermand not exacerbate opportunity cost, leading to discounting functions that are equivalently steep. An important aspect, then, in determining the presence, severity, and sign of the sign-effect is the nature of the outcome environment experienced. In conclusion, the “sign effect” (Frederick et al., 2002; Kalenscher & Pennartz, 2008; Loewenstein & Prelec, 1992), as the “magnitude effect” (Frederick et al., 2002; Kalenscher & Pennartz, 2008; Loewenstein & Prelec, 1992), is a consequence of experientially constrained reward rate maximization as conceptualized by TIMERR, not a flaw in rational decision-making.

#### 2.4.5 New perspective on the meaning of the terms in Equation (9)

In the above sections, we have treated $T_{\mathrm{ime}}$ as representing the total time an agent has spent within a session, and $a_{\mathrm{est}}$ as the ratio of the total reward achieved during this time to $T_{\mathrm{ime}}$. However, real animals face at least three major constraints that limit the validity of this interpretation: (1) their reinforcement environments are not stationary; (2) there is increasing computational and metabolic costs associated with integrating over a long time, and, (3) indefinitely long intervals without reward cannot be sustained by an animal (while maintaining fitness) even if they were to return the highest long-term reward rate (e.g., choice between 10,000,000 units of food in 100 days vs. 10 units of food in 0.1 day). Hence, we think that the duration of $T_{\mathrm{ime}}$ is more appropriately thought of as a “past integration interval” over which recent reward history $\left(a_{est}\right)$ is estimated, instead of the total time spent in an environment. The value of $T_{\mathrm{ime}}$ might in fact need to be adjusted to the current environment so as to make optimal decisions (see Section 4 for a longer discussion). Further, it must be pointed out that the past reward rate $\left(a_{est}\right)$ might not be as simply estimated as the ratio of the rewards accumulated over $T_{\mathrm{ime}}$, to $T_{\mathrm{ime}}$. This would imply that rewards obtained just beyond $T_{\mathrm{ime}}$ have zero contribution to the past reward rate whereas all rewards obtained within $T_{\mathrm{ime}}$ contribute fully. In fact, we have showed previously that if the updating of the past reward rate has to be done locally (without storing every past reward’s magnitude and time of receipt in memory), the past reward rate has to be calculated using an exponential weighting function (see Namboodiri et al., 2014) for further discussion). Hence, since $T_{\mathrm{ime}}$ is the *effective* duration over which past reward rates are estimated, we will refer to it from here on as the “past integration interval” and not the “look-back time” as in prior sections.

#### 2.4.6 Connection to experimental data

In this section, we review experimental data from a wide array of fields that can be systematized by our theory. For this purpose, we treat typical human and non-human animal experiments separately as typical human experiments differ from animal experiments in one fundamental way: humans are often given hypothetical rewards involving hypothetical delays. However, animals face real rewards requiring real investments of time. We will first cover non-human animal experiments. But before considering them separately, we will first discuss commonalities in animal and human experiments. In both groups, hyperbolic discounting has provided better fits to data than exponential discounting functions (e.g., Ainslie, 1974, 1975; Ainslie & Monterosso, 2003; Benzion et al., 1989; Calvert et al., 2010; Green et al., 1994; Holt et al., 2003; Kobayashi & Schultz, 2008; Rachlin et al., 1972; Thaler, 1981), as predicted by DUT (however for an exception, see Schweighofer et al., 2006). Our theory fits with this observation as Equation (9) predicts a temporal discounting function that is a hyperbolic discounting function minus a constant ($a_{\mathrm{est}}T_{\mathrm{ime}}$) that depends on the immediate reinforcement history.

Another finding that is consistently observed across humans and non-human animals is variability in the discounting steepness within and across individuals (e.g., Ainslie, 1974, 1975; Ainslie & Monterosso, 2003; Benzion et al., 1989; Blanchard et al., 2013; Calvert et al., 2010; Frederick et al., 2002; Green et al., 1994; Holt et al., 2003; Jimura et al., 2009; Kobayashi & Schultz, 2008; Myerson & Green, 1995; Odum, 2011; Pearson et al., 2010; Rachlin et al., 1972; Richards et al., 1999; Rosati et al., 2007; Shamosh et al., 2008; Thaler, 1981). While common accounts of such variability invoke differences in “personality” (or other psychological/neurological) traits (e.g., Bickel et al., 2007; Kalenscher & Pennartz, 2008; Madden & Bickel, 2010; Odum, 2011; Richards et al., 1999; van den Bos & McClure, 2013), we ascribe it a more functional meaning: variability across subjects reflects variability in the past integration interval, appropriate for the individuals’ respective reward environments. Relatedly, apparent differences between subjects may actually reflect differences in experienced past reward rate ($a_{\mathrm{est}}$ in Equation (9). Nonetheless, some variability across subjects may yet lie in subjects’ innate neural differences, independent of their environment or experience.

##### 2.4.6.1 Data from non-human animals

A fundamental prediction of Equation (9) is that the discounting steepness will depend on the past reward rate. This means that, for instance, when the duration between rewards is increased, animals should become more tolerant to delays, since the longer the duration between rewards, the lower the past reward rate and therefore, the lower the discounting steepness (Figure 6). Data from (Blanchard et al., 2013) discussed in Section 2.1 support this prediction. As mentioned above, they observed lower levels of discounting steepness when the post-reward delays (equal for both options in experiment 3) were increased. Since they wished to use this observation to advance a rate-maximizing equation such as Equation (5), they had to further assume that representations of post-reward delays are biased. As discussed earlier, this is not consistent with many other experimental results on time perception (e.g., Buhusi & Meck, 2005; Matell & Meck, 2004). A simpler explanation for their data, within a reward-rate maximizing framework is the one we’ve proposed. Another paper that observes a similar effect in a different task is Mazur and Biondi (2011). In their study, they found that the delay at which a delayed reward is treated as equivalent to a standard reward of fixed magnitude and delay, depended on the intertrial interval, again consistent with the effect of reducing past reward rates.

Another key prediction of Equation (9) is the “magnitude effect”, as shown in Figure 10. Before considering experimental evidence addressing whether animals show “magnitude effect”, note that in Figure 10, we assumed the past reward rate to be constant, or at least equal for both reward options. Is this assumption valid in interpreting results from experiments?

There are two kinds of experimental designs used to study “magnitude effect” in animals. One is an “adjusting-amount” or “titration” procedure. In this design, a standard reward option (with fixed delay and magnitude) is compared against an option with an immediate reward with varying magnitudes. The magnitude of the second option is adjusted depending on the animal’s previous choice. If the animals choose the second option, then its magnitude is decreased and vice-versa. Discounting functions obtained from such experiments did not show any difference in the indifference point—the magnitude at which both options are treated equivalently—for different magnitudes of the standard option (e.g., Calvert et al., 2010; Freeman et al., 2009; Green et al., 2004; Green & Myerson, 2004; Richards et al., 1997). Therefore, these authors conclude that there is no observable “magnitude effect”. Another kind of experimental design used to study “magnitude effect” in animals is a “concurrent chains task” (Grace, 1994, 1999; Grace et al., 2012; Kinloch & White, 2013; Ong & White, 2004). In this design, animals responded to concurrently available options (keys to peck, for instance) in a “choice phase” so as to access one of two reinforcement schedules (different rewards at differing delays) in an “outcome phase”. In the earlier versions of this experiment (Grace, 1999; Ong & White, 2004), the magnitude for both options was equal in one block. Once preferences at that magnitude were measured, observations were repeated for a different magnitude. In both the above types of experiments, it is clear that when the magnitude under study was large, so was the past reward rate. Looking back at Equation (9), it is evident that when the past reward rate is proportional to the reward magnitude, the effect of the reward magnitude cancels out such that there will be no “magnitude effect”. Interestingly, when the above authors repeated the “concurrent chains” design with a simultaneous change of magnitude and delay, they observed a “magnitude effect” in pigeons as well as humans (Grace et al., 2012; Kinloch & White, 2013). While this result has not been explained yet, it has a straightforward explanation in our framework: when both magnitude and delay are changed across reinforcement schedules, the past reward rate will not be simply proportional ($a_{est}$ would still show a positive correlation with r) to the reward magnitude, as when only the magnitude was changed. Hence, the $a_{est}/r$ term in the numerator of Equation (9) does not cancel out, revealing the “magnitude effect”.

It is important to point out that a descriptive model of discounting that has been recently proposed (Killeen, 2009) can indeed fit the “magnitude effect” data in Grace et al. (2012), along with an earlier descriptive model (Grace, 1994). Interestingly, Killeen’s additive utility model has mathematical similarities to Equation (9). Yet there is no explanation for *why* there is an additive term in this model. In our theory, a subtractive opportunity cost automatically emerges as a direct consequence of experientially informed rate maximization. Precisely due to this difference, our theory also predicts that in experimental designs where the subtractive opportunity cost term is directly proportional to the reward magnitude, there will be no observed “magnitude effect”. Such data cannot be explained by the additive utility model.

Another prediction of Equation (9) is the “sign effect”, as shown in Figure 11. This prediction is however, methodologically challenging in animals since it is difficult to create punishments of equal magnitudes as rewards. Nevertheless, it has strong support in human data, as will be discussed in the next section.

The most important prediction, however, of Equation (9) is as yet untested. This is the prediction that as the duration over which past reward rates are estimated increases (or decreases), the steepness of temporal discounting decreases (or increases). This will provide the direct falsifiable test of our theory. However, as discussed in the next section, some indirect evidence from humans supports the above prediction.

##### 2.4.6.2 Data from humans

As mentioned earlier, our theory, TIMERR, was developed in the context of optimal foraging, as we hoped to find an evolutionary argument for discounting. It is in fact derived from the same starting postulate as Optimal Foraging Theory—that animals evolved to maximize reward rates (the key difference being that while OFT holds that only future reward values matter, TIMERR uses the past to inform the maximization of reward; in some sense, an algorithm for infinite-time-horizon maximization of reward rates, as postulated in OFT, can be thought of as TIMERR with $T_{\mathrm{ime}}=\infty$). Hence, the theory was developed assuming that tasks faced by animals involve real rewards which require real investments in time. Crucially, all animal tasks that we are aware of require the animals to invest the delay solely for the purpose of collecting an offered reward. In other words, in animal tasks, they cannot go about their daily lives seeking other opportunities for reward while waiting for the promised reward to become available. This is, however, different for typical human tasks (see Frederick et al., 2002; Kalenscher & Pennartz, 2008 for a review). In typical human tasks, one is asked hypothetical questions involving hypothetical delays. Let us consider an example: “what would you prefer: $10 in an hour or $20 in four hours? After the chosen delay expires, we will come find you and pay you the amount”. From anecdotal experience, we have observed that most people prefer $20 in four hours. How does this vary from typical animal tasks? To see this difference, let us now consider a different question: “what would you prefer: $10 for waiting in line for an hour or $20 for waiting in line for four hours? If you wait for the delay, the reward delivery is certain”. Interestingly, the choice in this question is typically the former: most people do not want to invest the *real* four hours for obtaining just $20. Even more interesting was the observation that anyone that tended to still favor the $20 was a (poor) graduate student, or currently unemployed. This too, makes sense. If one’s hourly salary is being cut during the wait time, a rich CEO would never wait four hours for $20, whereas a graduate student might gladly do so because his/her opportunity cost for waiting is close to zero. This experiment, though anecdotal, immediately makes it intuitive that animal experiments are different from typical human experiments involving hypothetical questions. It also intuitively explains why it is important for animals to include opportunity costs in their decisions. TIMERR goes beyond this simple realization only in deriving that the effective time interval over which opportunity costs are calculated directly determines the steepness of temporal discounting, in addition to the opportunity cost itself.

In light of this realization that opportunity costs are not directly enforced in typical human tasks, how can TIMERR explain such data? Does Equation (9) apply to such decisions? We reason that the mathematical form of Equation (9) would still apply to such decisions but the meaning of the terms might be different. Specifically, we believe that $T_{\mathrm{ime}}$ would still be a past integration interval, but instead of it being determined by the experience of the subject, it might be determined by the hypothetical choice at hand. For instance, when one considers choices involving delays of seconds versus choices involving delays of hours, the past integration interval would adjust flexibly so as to provide an appropriate time frame for the question at hand. Perhaps this underlies the observation that when average delays are long, temporal discounting is correspondingly shallow, compared to when the average delays are short (e.g., Carter et al., 2010; Frederick et al., 2002; Jimura et al., 2009; Kable & Glimcher, 2007; Kalenscher & Pennartz, 2008; Loewenstein & Prelec, 1992; Luhmann et al., 2008; McClure et al., 2004; Rosati et al., 2007; Schweighofer et al., 2006).

The numerator of Equation (9), on the other hand, has a subtractive term which reflects the opportunity cost of waiting. Since there is no real waiting required, one might suppose that opportunity costs may not exist in such decisions. However, the subtractive term in the numerator of Equation (9) could arise due to two possible reasons: 1) even though such choices are typically one-shot, it might be implicitly assumed that the choices will repeat, such that an opportunity cost based on the presented options (e.g., average reward rate of the presented options) is automatically included in the decision, or alternatively, 2) humans could incorporate a model of linear risk into such decisions.

In the former possibility, the subjective value of a delayed reward would still be given by Equation (9), but with the $a_{\mathrm{est}}$ term representing the average reward rate modeled based on the current environment (i.e., current options). A prediction of this account would be that every option presented on a given choice will affect the decision; in a choice between two options, adding a third decoy option worse than both should have an effect on the choice. A similar observation (though not in intertemporal decisions) is commonly known as the “decoy effect” or the “asymmetric dominance effect” in marketing (e.g., Huber, Payne, & Puto, 1982), wherein adding a decoy option (inferior in all respects to one option but not easily comparable to the other) automatically shifts preference towards the option that is superior to the decoy. For a longer discussion on how decisions between options are made in relation to the environment, see Ariely (2008).

To explain the second possibility of a linear risk, let us consider an example: “you are offered a bag of M&M’s candy containing 100 candies, available to you after 15 minutes. However, during the 15 minutes, the bag is left open on a table in the hallway. How many candies would you expect to receive after 15 minutes?” If one assumes that there is a constant probability of 0.5 that everyone walking by the hallway will pick a candy each, and one expects 20 people to walk by the hallway in 15 minutes, the expected number of candies will be: 100 – 0.5*20*1 = 90. If the bag were left out for a time long enough that at least 200 people are expected to pass by, the number of candies left would be zero.

Thus, the simplest model of risk involved in delaying a reward is that the offered reward reduces in magnitude at a constant linear rate over time, i.e.

(10) $$\frac{dr(t)}{dt}=-k$$

If humans do include such a risk model in their decisions, the expected reward that can be collected after waiting the given delay of t will be r(t)=r-kt. If they simply calculate the net expected reward rate by dividing this quantity by the past integration interval plus the delay t (just like in the earlier treatment of TIMERR), their subjective value will look like Equation (9), with the linear opportunity cost term $\left(a_{\mathrm{est}}t\right)$ being replaced by kt, i.e.

(11) $$\begin{array}{r} SV(r,t)=\frac{r\;-\;kt}{1\;+\;\frac{t}{T_{\mathrm{ime}}}};t<\frac{r}{k}\\ 0\;;t\geq \frac{r}{k} \end{array}$$

Hence, the mathematical predictions from Equation (9) like “magnitude effect” and “sign effect” would still hold.

We do not know why a risk model such as the one envisaged in Equation (10) would be applied in these hypothetical questionnaire tasks. Nevertheless, a similar risk model has been assumed in a recent descriptive model of discounting (Killeen, 2009). Also, see our previous paper (Namboodiri et al., 2014) for more complex models of risk. Mathematically, the two possibilities mentioned above are equivalent and result in the same predictions presented earlier.

Numerous reports provide evidence of the “magnitude effect” (Benzion et al., 1989; Frederick et al., 2002; Green et al., 1994; Green et al., 1997; Thaler, 1981) and the “sign effect” (Benzion et al., 1989; Frederick et al., 2002; Thaler, 1981) in humans. This has also been repeated using real rewards, instead of hypothetical rewards. In fact, a recent paper using a task typically done by animals (“concurrent chains task” mentioned in Section 2.2.11.1) in humans choosing between hypothetical rewards also showed a clear “magnitude effect” (Kinloch & White, 2013). Another prediction of TIMERR is that the size of the “sign effect” will be larger for smaller magnitudes (see *Consequences of the Discounting Function* in the Appendix of Namboodiri et al., 2014). This too is supported by experiments (Benzion et al., 1989; Loewenstein & Prelec, 1992; Thaler, 1981). Further, Equation (9) predicts that a magnitude-like effect for losses (in net rewarding environments) will be in the opposite direction as that for gains, i.e. losses of larger magnitudes will be discounted steeper than losses of smaller magnitudes. This was recently tested by Hardisty et al. (2012) and found to be true.

Yet another prediction similar to the above effects is that losses will be treated differently depending on their magnitude (see *Consequences of the Discounting Function* in the Appendix of Namboodiri et al., 2014). Specifically, a smaller loss (a loss smaller in magnitude than the magnitude of the accumulated reward) will become even more of a loss when delayed, whereas a larger loss becomes less of a loss with additional delay. Such a differential treatment of losses is also widely observed in experiments (Benzion et al., 1989; Berns et al., 2006; Loewenstein, 1987; Mackeigan et al., 1993; Mischel et al., 1969; Redelmeier & Heller, 1993; Yates & Watts, 1975). The above experiments rationalize this result as resulting from increased “anticipation” of losses (e.g., see Loewenstein, 1987). However, we do not have to make such *ad-hoc* justifications; it is a natural consequence of reward rate maximization, as in our theory.

Another “anomalous” observation (from the perspective of DUT) is the “delay-speedup effect” (Benzion et al., 1989; Loewenstein, 1988; Loewenstein & Prelec, 1992). This refers to the observation that subjects expect more compensation for delaying receipt of a reward (from $t_{1}$ to $t_{2}$) than they are willing to pay to speed it up by that same amount (from $t_{2}$ to $t_{1}$). There are two potential explanations for this phenomenon. One explanation, as previously noted by (Killeen, 2009) is that it could simply be a consequence of recursive discounting, i.e. the subjective value of a reward delayed from $t_{1}$ to $t_{2}$ is the subjective value of the reward at $t_{1}$ discounted again by the additional delay of $t_{2}-t_{1}$. Thus, a reward expected at $t_{1}$ when delayed to $t_{2}$ has a subjective value lower than a reward expected at a delay of $t_{2}$. We will not mathematically prove this property here for Equation (9) since it is straightforward. One problem, however, with this account (and the one presented in (Killeen, 2009)) is that if $t_{1}$ were zero, there is no predicted “delay-speedup effect”, even though experiments clearly demonstrate an effect even when $t_{1}=0$ (Benzion et al., 1989; Loewenstein, 1988; Loewenstein & Prelec, 1992). Another potential explanation is that whenever subjects are told that they have “received a reward at time $t_{1}$”, they automatically incorporate the receipt of that reward into the risk term in Equation (11). This is more easily understood with the M&M’s example. The “delay” experiment goes thusly: “imagine you have received 100 M&M’s. How many more M&M’s should I give you such that you are willing to delay receipt by 15 minutes?” Going back to the calculation above, if you expect that in 15 minutes, the *actual number* of M&M’s you receive is 90 (since 20 people take one each at 0.5 probability), you will require compensation for this assumed reduction as well. On the other hand, the “speedup” experiment goes thusly: “imagine you will receive 100 M&M’s in 15 minutes. How many are you willing to pay so as to receive it immediately?” When one evaluates this question, it makes sense to assume that it is *certain* that the 100 M&M’s exist after 15 minutes (because it has not been left out in the hallway). Hence, in speeding up its receipt, one only needs to pay for the pure temporal cost (and not the linear risk cost). Of course, further experiments will be needed to test this hypothesis. Note also that both of the above arguments make it clear that delaying a reward in multiple steps by short delays causes it to decay faster than delaying it by the total amount all at once. This effect has been previously observed and labeled as “subadditivity” (Read, 2001).

In sum, most human discounting tasks differ from animal tasks in a crucial aspect: humans do not have to wait the corresponding delays. Consequently, the opportunity cost term in TIMERR should not directly apply to these tasks. However, assuming that humans employ a simple model of risk (constant decay) in such decisions — similar to an assumption made previously in a successful descriptive model (Killeen, 2009) — preserves all of the mathematical qualities of the subjective value of a delayed reward as derived by TIMERR. Evidence collected across numerous experiments (see Kalenscher & Pennartz, 2008; Loewenstein & Prelec, 1992; Madden & Bickel, 2010 for reviews) support these predictions. Recently, there have been attempts to test humans on discounting tasks similar to animal tasks, requiring them to wait out the delays for real rewards (e.g., Reynolds & Schiffbauer, 2004). More work is needed to know whether behavior in this task will reproduce the above results (see Jimura et al., 2009; Krishnan-Sarin et al., 2007; Melanko et al., 2009; Reynolds, 2006; Reynolds et al., 2008; Shiels et al., 2009; Smits et al., 2013).

## 3 Connection Between Theories of Intertemporal Decision-Making and Time Perception

As mentioned early in the Introduction, it is only natural that time perception and intertemporal decision-making go hand-in-hand since the latter requires the former. It is also then intuitive to assert that the former is more fundamental than the latter. In this view, mechanisms of time perception evolved under their own evolutionary pressures, whereas intertemporal decision-making was only as good as animals’ ability to perceive the delays to reward. Hence, all prior attempts to connect intertemporal decision-making and time perception have assumed time perception to be the more fundamental of the two processes (e.g., Bateson, 2003; Cui, 2011; Gibbon et al., 1988; Kacelnik & Bateson, 1996; Kim & Zauberman, 2009; Lapied & Renault, 2012a; Meck, 2003; Ray & Bossaerts, 2011; Takahashi, 2005, 2006; Takahashi et al., 2008; Zauberman et al., 2009).

The *sine-qua-non* of theories of time perception is their treatment of Weber’s law (see Allman et al., 2014; Bateson, 2003; Gibbon, 1977; Gibbon et al., 1997; Killeen & Fetterman, 1988; Matthews & Meck, 2014; Matell & Meck, 2000). Weber’s law is easily understood by considering an example. Imagine that one is tasked with finding the longer rod among two iron rods placed in parallel from just a glance; no measurements or realignments are allowed. If one of them were a meter and the other two, the difference should be as clear as night and day. However, if they are a hundred meters and a hundred and one meters long, this will be considerably more difficult, even though the difference is still one meter. Essentially, Weber’s law states that the difference between two quantities is judged relative to the quantities themselves. Weber’s law also appears in timing: it is easier to discriminate one second from two seconds than to discriminate one hundred seconds from one hundred and one seconds. A mathematical statement of Weber’s law for timing goes even further. It states that the precision of perceiving an interval decreases in direct proportion to the interval; in other words, the error in perception of an interval grows linearly in proportion to the interval. This relationship is more commonly known as scalar timing, and has been observed repeatedly in experiments for humans and non-human animals (e.g., Allan & Gibbon, 1991; Cheng & Meck, 2007; Church & Gibbon, 1982; Gibbon, 1977, 1992; Gibbon et al., 1984, 1997; Gibbon & Church, 1981; Lejeune & Wearden, 2006; Matthews & Meck, 2014; Meck & Church, 1987; van Rijn et al., 2014; Wearden & Lejeune, 2008).

Before we consider the different theories of time perception and how they explain scalar timing, we would like to explain how scalar timing can explain non-stationary time preferences. Recall that the most well-established result on intertemporal decision-making is that time preferences are non-stationary. Let us reconsider the two example questions we provided earlier to illustrate this point: “which would you prefer: $100 now or $105 in a month?” and “which would you prefer: $100 in a year or $105 in a year and one month?” Considering the above questions from the perspective of scalar timing, it must be immediately clear that the month of difference in the first question is “subjectively longer” than the month in the second question (of course, when the numbers are specified, they should be treated as mathematically equivalent, but animals must estimate such durations from experience). So essentially, even though the interval between both rewards is constant, the further away the rewards are from the decision, the harder it is to discriminate between the two delays. Hence, as the delays are perceived to be more and more similar, the tendency to pick the larger reward should increase. Thus, scalar timing can lead to non-stationary time preferences.

The above argument is the essence of numerous prior attempts to connect theories of time perception to theories of intertemporal decision-making (Bateson, 2003; Cui, 2011; Gibbon et al., 1988; Kacelnik & Bateson, 1996; Stephens, 2002; Zauberman et al., 2009). In fact, it has also been shown that if the perception of time were logarithmic, which is consistent with scalar timing, an exponential discounting in subjective time is mathematically equivalent to a hyperbolic discounting in real time (Takahashi, 2005; Zauberman et al., 2009). Similar explanations also exist using other non-linear representations of subjective time (Ray & Bossaerts, 2011). Thus, it has been argued that the reason for non-stationary intertemporal preferences is because of imperfections in time perception (Bateson, 2003; Cui, 2011; Gibbon et al., 1988; Kacelnik & Bateson, 1996; Kim & Zauberman, 2009; Lapied & Renault, 2012a; Ray & Bossaerts, 2011; Takahashi et al., 2008; Takahashi, 2005, 2006; Zauberman et al., 2009). For other possible connections between time perception and intertemporal decision-making see Balci et al. (2009, 2011) and Heilbronner and Meck (2014).

Now, let us consider the different theories of time perception. All the theories considered here pertain to interval timing, i.e. timing in the range of seconds to minutes. Further, we will only consider two of the most popular theories of timing, as our major goal here is in studying the relationship between intertemporal decision-making and time perception. For a more thorough review of the models of timing, see the many reviews and primary articles that have been written on this topic (e.g., Ahrens & Sahani, 2011; Allman & Meck, 2012; Allman et al., 2014; Almeida & Ledberg, 2010; Buhusi & Meck, 2005; Buonomano & Karmarkar, 2002; Buonomano, 2007; Gallistel & Gibbon, 2000; Gavornik et al., 2009; Gibbon et al., 1997; Grossberg & Schmajuk, 1989; Hass et al., 2008; Hass & Herrmann, 2012; Ludvig et al., 2008; Luzardo et al., 2013; Machado, 1997; Matell & Meck, 2000, 2004; Mauk & Buonomano, 2004; Meck, 1996; Meck & Benson, 2002; Merchant et al., 2013; Miall, 1989; Shankar & Howard, 2012; Simen et al., 2011, 2013; Teki et al., 2011; Treisman, 1963; Wackermann & Ehm, 2006).

The most popular theory of timing is the Scalar Expectancy Theory (SET) – also known as scalar timing theory (Church, 2003; Gibbon et al., 1984; Gibbon, 1977; Matell & Meck, 2000). This theory proposes that there are three different modules working together to time an interval. The first stage is a pacemaker that produces a continuous pulse train that is gated on to a second, accumulator, stage upon receipt of a signal to time. The accumulator counts the number of pulses until the end of the interval being timed. The total number of accumulated pulses is then compared against a representation of that interval stored in a reference memory stage. Crucially, the comparison is based on a ratio-rule so as to achieve scalar timing. Such ratio comparisons are commonly observed in many timing tasks (Gibbon & Fairhurst, 1994; Gibbon et al., 1997; Gibbon, 1992). The most important source of variability in timing (among variability in clock speed, clock reliability, accumulation, comparator and memory) according to SET is the variability introduced when a current duration in working memory is compared to a remembered duration (Gibbon et al., 1984).

A major competitor to SET is the Behavioral Theory of Timing (BeT) (Killeen & Fetterman, 1988). BeT, too, effectively uses a similar pacemaker-accumulator system. BeT states that signals that instruct timing onset result in a series of “adjunctive behavioral states”—a stereotyped sequence of psychological states—that indicate the passage of time. According to BeT, animals tell time by knowing the position of their current adjunctive state in relation to the sequence. In the simplest approximation, the transition time between states was assumed to be exponentially distributed as resulting from a Poisson clock, thereby making the arrival time of a given state, gamma distributed. The core assumption of BeT is that the rate of the Poisson accumulation is proportional to the reinforcement rate. As most timing tasks confound reinforcement rate with the target duration (since in many tasks, rewards are received only at the end of the intervals to be timed) (e.g., Killeen, 1975; Killeen & Fetterman, 1988), the above assumption means that the standard deviation of the distribution of a given adjunctive state is proportional to the period of food delivery, thus resulting in scalar timing (Bizo et al., 1997; Killeen & Fetterman, 1988).

We are not going to review the merits and demerits of the above theories or any of the alternate theories of timing here (e.g., Ahrens & Sahani, 2011; Almeida & Ledberg, 2010; Creelman, 1962; Gallistel & Gibbon, 2000; Gavornik et al., 2009; Grossberg & Schmajuk, 1989; Hass et al., 2008; Karmarkar & Buonomano, 2007; Ludvig et al., 2008; Luzardo et al., 2013; Machado, 1997; Matell & Meck, 2000, 2004; Meck, 1996; Miall, 1989; Shankar & Howard, 2012; Simen et al., 2011; Teki et al., 2011; Treisman, 1963; Wackermann & Ehm, 2006). These can be found in Buhusi and Meck (2005), Gibbon et al. (1997), Matell and Meck (2000, 2004), Merchant et al. (2013), and Simen et al. (2013). However, a central consideration missing in the above is that none of these models really attempts to answer the question of *why* Weber’s law is (or should be) true; they only address *how* Weber’s law is generated. Clearly, an ideal timing system, free of any noise, should be able to precisely time any duration. A first-order deviation from this ideal system would have a constant error in timing at any duration — not an error that increases in proportion to the duration. So what could be the reason behind a scalar increase in noise?

One possible solution is to postulate that animals’ timing can only be as good as the neurons that help them to time. Since we know that neurons are inherently noisy information processors, timing behaviors will reflect the noise-duration relationship that neurons produce (see Oprisan & Buhusi, 2014). Let us now consider the simplest assumption of noisiness in neuronal firing, i.e. that they fire according to a Poisson process of rate λ. Assume that timing of an interval is done by counting the number of spikes: the moment the target number of spikes (say n) has been emitted, the interval is deemed to have expired. Hence, the expiration of an interval is treated as the arrival time of the $n^{\mathrm{th}}$ spike. For a Poisson distribution, the arrival time of the $n^{\mathrm{th}}$ spike has an Erlang distribution (special case of a gamma distribution) with a mean and standard deviation expressed as:

(12) $$\mu =\frac{n}{\lambda };\sigma =\frac{\sqrt{n}}{\lambda }$$

The corresponding $C_{v}$ (coefficient of variation = standard deviation/mean) is given by

(13) $$C_{v}=\frac{1}{\sqrt{n}}$$

If the firing rate of the Poisson process is represented as λ, the number of spikes required to time a target interval of t is simply n=tλ. Thus, the $C_{v}$ can be re-written as $\frac{1}{\sqrt{t\lambda }}$. Therefore, it is clear that for a Poisson process to produce scalar timing (constant $C_{v}$), its rate has to be inversely proportional to the interval being timed (i.e. tλ=constant). To be sure, the above treatment is the exact mathematical equivalent of BeT. However, the difference is in the meaning of the variables. While BeT’s Poisson process is an abstract sequence of behavioral states, the above Poisson process is a spike train of a neuron. Owing to this critical difference, BeT had to assume that the rate of the Poisson process is inversely proportional to the reinforcement density (which is in turn inversely proportional to the target time, in typical tasks) so as to obtain a constant tλ. This assumption was not borne out empirically (Bizo et al., 1997). But the mathematical elegance of BeT is captured by the above treatment of a Poisson neural spike train.

To summarize, here is a simple neural model for timing that produces scalar timing: a Poisson spike train is initiated with a rate inversely proportional to the time interval to be timed. The interval is read out as the time to fire a constant threshold of spikes. Let us now actually calculate some numbers to see if this model is neurally plausible. Typical $C_{v}$ values are around 0.1–0.5 in animals (e.g., Matell et al., 2003, 2004, 2006). From Equation (13), the number of spikes for the threshold, n, is approximately 11 (considering a $C_{v}$ of 0.3). Since n=tλ, this means that in order to time an interval of 11s, this neuron has to spike at 1Hz, and at 0.1Hz for an interval of 110s. This firing rate is prohibitively low to be neurally plausible. Hence, the above simple neural model which is mathematically equivalent to BeT cannot be implemented by single neurons.

**Figure 12.**
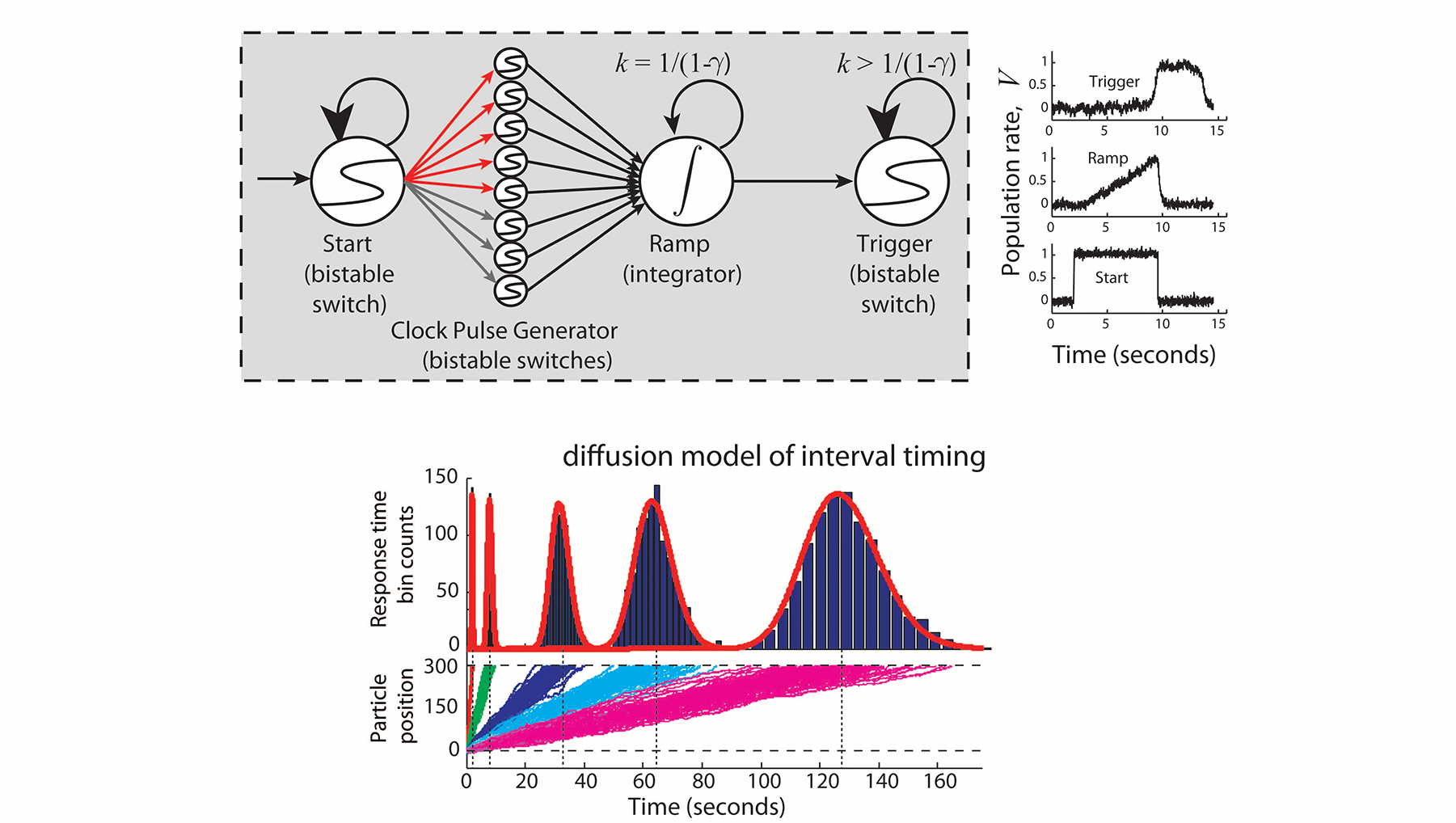
A neural accumulator circuit that implements the simple mathematical argument (similar to BeT) presented in Section 3, modified from Figure 1 in Simen et al. (2011). There are four different modules in the model. Bistable switches turn on upon timing onset (“start”) to produce clock speeds adjusted to the target interval (“clock pulse generator”). An “integrator” produces a linearly rising ramp with the passage of time. The moment the ramp hits a threshold, a “trigger” turns on indicating the lapse of the target interval so as to produce the corresponding behavior. Simulations of the model are shown below to show the different clock speeds producing an inverse-Gaussian distribution of timed intervals (Simen et al., 2011).

However, one could imagine the above model to be implemented by a network of excitatory and inhibitory neurons working so as to produce a balanced integration of Poisson-like spikes. This is exactly what was done in 2011 by Simen et al. (Simen et al., 2011). They showed that under some simple assumptions, a drift-diffusion accumulator model of timing comprising Poisson neurons connected into balanced excitatory and inhibitory networks can work similarly to the mathematical model presented above. This is shown in Figure 12. In this conception, the mathematics of BeT is a special case when there is no inhibitory input. We will not go into a more detailed review of this model, other than to point out that while it retains most of the mathematical elegance of BeT (and the treatment above), it differs in some quantitative details; the distribution of the timed interval is inverse-Gaussian and not a gamma distribution (Simen et al., 2011).

Hence, according to the view of the above theories, scalar timing is a consequence of noisy information processing by neurons. It must be emphasized that the above models and theories of timing do not postulate any direct correlations between time perception and intertemporal decision-making. Recent experimental evidence, however, suggests otherwise (Baumann & Odum, 2012; Wittmann et al., 2007; Wittmann & Paulus, 2008). In fact it was found that in general, individuals with better perception of time were more tolerant to delay (e.g., Baumann & Odum, 2012; Wittmann & Paulus, 2008) (for a more detailed discussion of these results, see Section 4). This result is not consistent with the proposition earlier in this section that Weber’s law underlies non-stationary time preferences. This is because as the perception of time becomes better and the delays between two options become better discriminated, the tendency to pick the smaller reward should increase (compared to when the delays are judged to be similar). The above argument clearly predicts a reduced tolerance to delay with better perception of time, in contrast to experimental data.

In our theory, we approached the problem of time perception entirely differently. In fact, we argued that intertemporal decision-making is the more fundamental of the two, completely opposite to all the prior attempts to connect intertemporal decision-making and time perception (e.g., Bateson, 2003; Cui, 2011; Gibbon et al., 1988; Kacelnik & Bateson, 1996; Kim & Zauberman, 2009; Lapied & Renault, 2012a; Ray & Bossaerts, 2011; Takahashi et al., 2008; Takahashi, 2005, 2006; Zauberman et al., 2009). We postulated that time is subjectively represented in such a way that the subjective representation of reward rate (subjective value per unit subjective time) is an accurate reflection of the true change in expected reward rate and/or reward value (cf., Lake & Meck, 2013; MacDonald et al., 2012; Meck, 1988, 2006; Meck et al., 2012; Yin & Meck, 2014).

Using the same symbols as in Section 2.2, this postulate can be expressed as

(14) $$\frac{SV(r,\;t)}{ST(t)}=\frac{r}{t}-a_{est}$$

**Figure 13.**
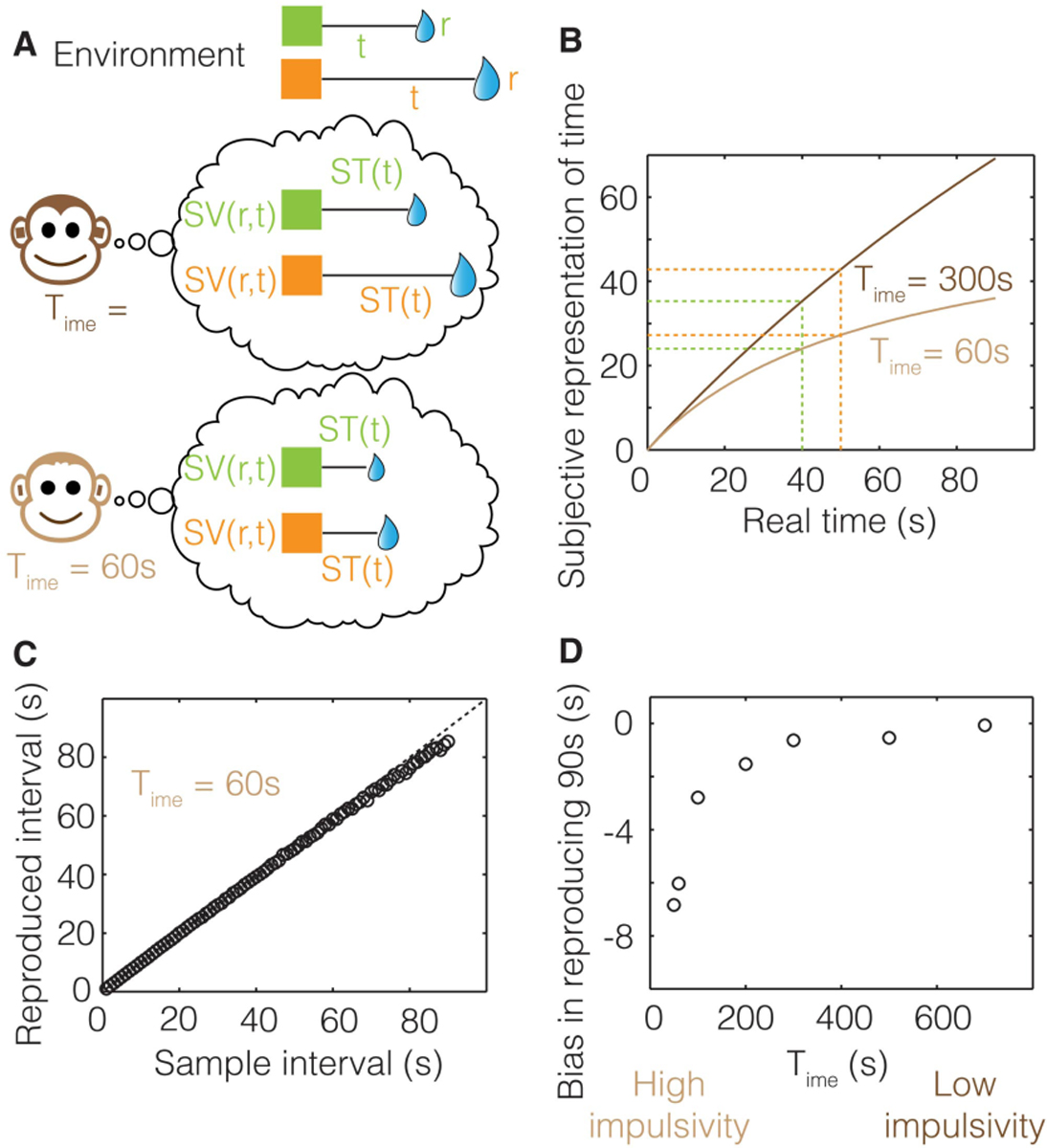
Reprinted from Namboodiri et al. (2014). A. Two illustrative animals with different values of the past integration interval ($T_{\mathrm{ime}}$) are shown, along with their respective subjective representations of the rewards. B. The subjective representation of time function as expressed in Equation (15) is plotted for both cases, indicating that the ability to discriminate between the subjective representations of 40 and 50 seconds is higher for the monkey with $T_{\mathrm{ime}}=\mathrm{300}s$. C. Results from a simulated time reproduction task are shown using an accumulator model as described in Namboodiri et al. (2014), demonstrating the underproduction of long intervals. D. The underproduction of intervals, interpreted commonly to result from a faster “clock”, is more pronounced when $T_{\mathrm{ime}}$ is low. Hence, individuals with low values of $T_{\mathrm{ime}}$ will appear as if their internal “clocks” are faster.

Here, ST(t) is the subjective representation of the true delay to reward t. Solving for the subjective time, we get the following simple expression

(15) $$ST\left(t\right)=\frac{t}{1\;+\;\frac{t}{T_{ime}}}$$

where $T_{\mathrm{ime}}$ is the past integration interval, as mentioned in Section 2.2. It must be emphasized that the subjective representation of the delay is not the subjective report of the delay; it is how the interval is represented on a subjective neural scale. Figure 13 shows a plot of ST(t) with respect to the actual delay, t. From the plot, it is clear that the ability to discriminate between intervals decreases as they increase. Further, low values of $T_{\mathrm{ime}}$ lead to underproduction of time intervals in a simulated time reproduction task (Figure 13C,D), *appearing* as if an internal “clock” is running faster. To see the details of how this accumulator model works, see Namboodiri et al. (2014), but an intuitive explanation for why there is an underproduction of intervals for low $T_{\mathrm{ime}}$ is as follows: during the production phase of the reproduction task, the time is judged to have elapsed when the accumulator hits for the *first* time the remembered threshold (from the estimation phase). Since the more non-linear the dependence of ST(t) on t (i.e. the lower the value of $T_{\mathrm{ime}}$), the more the chance that the accumulation will hit the threshold earlier (due to the noise in accumulation), and hence, the higher the bias towards an earlier time.

**Figure 14.**
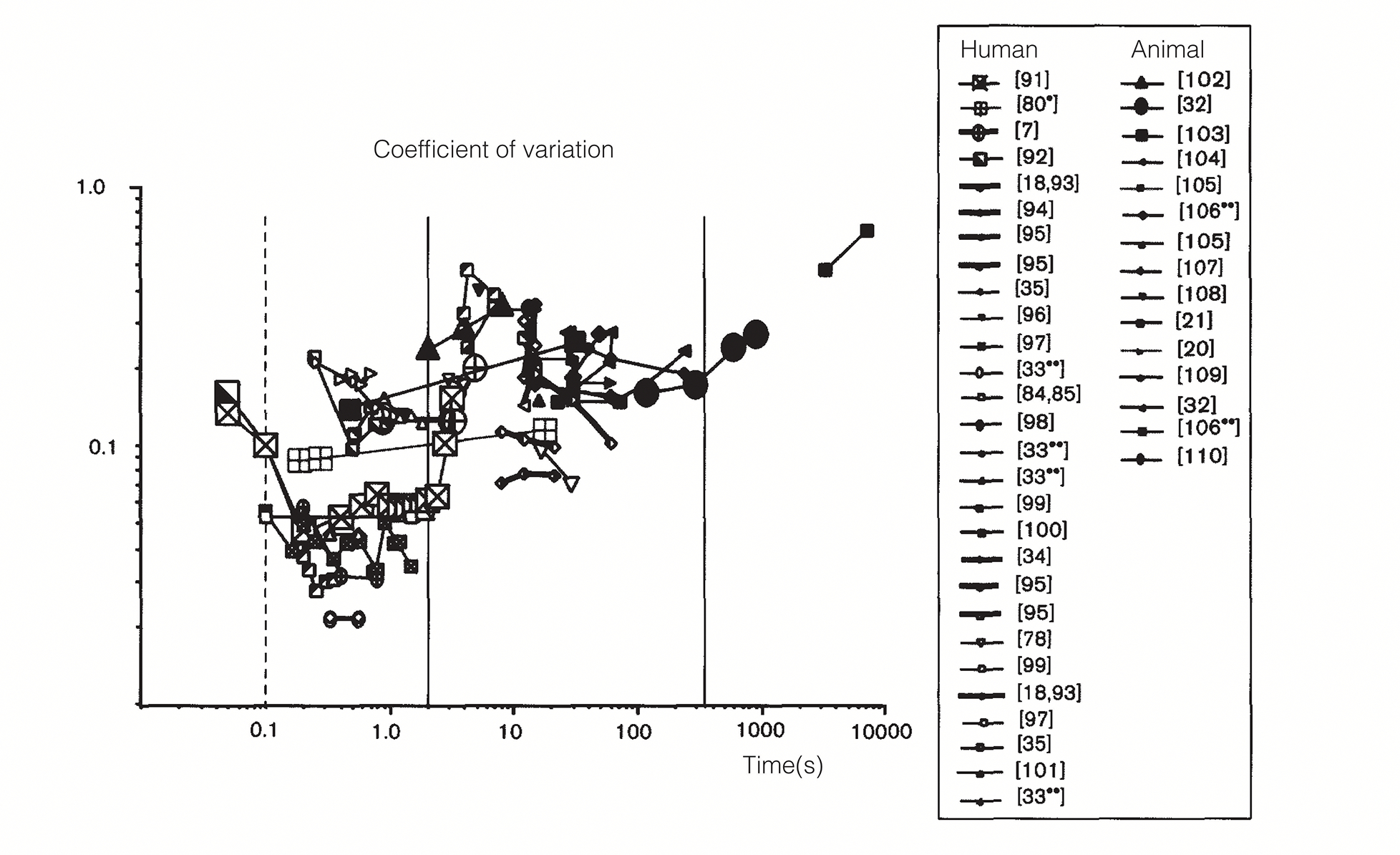
Coefficient of variation across different timing tasks is plotted as a function of the interval being timed. Adapted from Figure 3 in Gibbon et al. (1997).

We showed previously (Namboodiri et al., 2014) that a noisy accumulator model implementing the above equation leads to the following expression of $C_{v}$

(16) $$Cv\approx k\left(1+\frac{t}{T_{\mathrm{ime}}}\right)+\frac{c{\left(1\;+\;\frac{t}{T_{\mathrm{ime}}}\right)}^{2}}{t}$$

where c is a constant additive noise in a memory process and k represents the accumulation noise. If c=0, the above equation implies a constant $C_{v}$ plus a deviation that decreases with the past integration interval. As the past integration interval becomes larger and larger, the coefficient of variation is closer and closer to a constant. In other words, timing becomes more and more scalar the larger the value of the past integration interval (and larger the tolerance to delay in intertemporal decisions). Crucially, Equation (16) predicts quantitative deviations from scalar timing, depending on the interval being timed and the past integration interval. In fact, a review of thirty four studies in 1997 by the creator and proponents of scalar timing (Gibbon et al., 1997) observed a $C_{v}$ that increased with the duration being timed (see Figure 14), as predicted by Equation (16) (assuming constant $T_{\mathrm{ime}}$ across these experiments). For other studies showing an increase in $C_{v}$ at long durations, consistent with Equation (16), see Bizo et al. (2006), Lejeune and Wearden (1991), Zeiler (1991), Zeiler and Hoyert (1989), and Zeiler and Powell (1994), but also see Lewis and Miall (2009) for the opposite pattern. Nevertheless, it must be pointed out that Equation (16) results from specific assumptions (e.g., Poisson-like noise in accumulation) about the neural implementation of Equation (15). It is possible that more detailed and realistic neural implementations might differ from the approximation expressed in Equation (16).

Another key feature of temporal discrimination tasks is the point of subjective equality (PSE)—the duration which is judged to be equidistant between two intervals to be discriminated. SET predicts PSE to be at the geometric mean of the two intervals (Allan & Gibbon, 1991; Gibbon, 1977; Gibbon et al., 1984). This prediction received considerable experimental support (e.g., Allan & Gibbon, 1991; Church & Deluty, 1977; Gibbon, 1986; Melgire et al., 2005; Penney et al., 2000; 2008; Platt & Davis, 1983) and has been proposed to either result from ratio comparisons using linear subjective time or from logarithmic subjective time (Gibbon & Church, 1981). However, some experiments have shown deviations from the geometric mean (e.g., Kopec & Brody, 2010; Wearden, 1991). In fact, in a re-analysis of some earlier data (claiming PSE at geometric mean) using a more precise model, (Killeen et al., 1997) found that the PSE was found to vary between the harmonic mean and the arithmetic mean. Our theory also predicts that the PSE will be between the harmonic and arithmetic mean depending on the past integration interval. The lower the past integration interval, the more non-linear the representation of time becomes and the closer the PSE gets to the harmonic mean. When the past integration interval is very large, the PSE instead approaches the arithmetic mean. It must be pointed out that we have not attempted to quantitatively fit the PSE observed in previous studies because of the difficulty in knowing the value of $T_{\mathrm{ime}}$ across these studies. However, for a different model that attempts to systematically explain variance in the PSE, see Kopec and Brody (2010). See also Balci et al. (2011) for an interesting perspective on how differing abilities to time would dictate different optimal PSE values.

In sum, prior theories of time perception treat time perception as independent of intertemporal decision-making. Attempts to connect both have always treated time perception as more fundamental, implicitly assuming that mechanisms of time perception evolved under their own selective pressures. The most popular of these models are accumulator models, in which scalar timing is assumed to result from different sources of neural variability connected to the accumulation process (e.g., accumulation variability, pacemaker variability, memory variability etc.). However, in TIMERR theory, we postulate that intertemporal decision-making is more fundamental, i.e. that animals evolved to maximize reward rates and that scalar timing is a result of representing time subjectively so that the subjective reward rate accurately represents the change in expected reward rate (Equation (14)). This model is able to explain some experimentally-observed deviations from Weber’s law (but fails to explain some others) as well as individual variability in points of subjective equality in temporal discrimination experiments. More crucially, however, according to our theory, time perception is fundamentally linked to intertemporal decision-making: the higher the tolerance to delays, the better the perception of time. Ours is the only current mathematical theory that can explain such experimentally observed correlations systematically (Barkley et al., 2001; Barratt, 1983; Bauer, 2001; Baumann & Odum, 2012; Berlin et al., 2004; Berlin & Rolls, 2004; Bickel & Marsch, 2001; Dougherty et al., 2003; Heilbronner & Meck, 2014; Levin et al., 1996; Pine et al., 2010; Reynolds & Schiffbauer, 2004; van den Broek et al., 1992; Wittmann et al., 2007; Wittmann & Paulus, 2008).

To revisit, the reason why we treated decision-making as fundamental is that while decision-making can be clearly treated within the optimization problem of maximizing reward rates, there is no such well-defined optimization problem for time perception (in fact, the most optimal model would have a constant precision, independent of the duration represented). Interestingly, while previous models that connect intertemporal decision-making and time perception treat the psychophysical observations of time perception (scalar timing) as a fundamental postulate, we are able to derive approximate scalar timing from reward rate maximization. We would like to point out that the fact that such a connection can be made does not prove that time perception indeed evolutionarily followed the need to maximize reward rates, nor does it mean that every instance of time perception requires decision-making (making intertemporal decisions, of course, necessitates the measurement of time). It only suggests that maximizing reward rates might underlie the evolutionary origin of the psychophysics of time perception.

In the next section, we focus on how variability in the past integration interval leads to corresponding variability in intertemporal decision-making and time perception.

## 4 Impulsivity in the Domain of Time

As discussed in Section 2.2 and Section 3, our theory postulates that the duration over which the past reward rate is estimated ($T_{\mathrm{ime}}$) directly determines the tolerance to delays in intertemporal decision-making (i.e. the steepness of temporal discounting) and the non-linearity of time perception. Hence, a fundamental question is how the past integration interval is determined. We have previously listed a set of qualitative arguments on this issue (see “*Effects of Plasticity in the Past Integration Interval*” in Appendix of Namboodiri et al., 2014) and here, we will present a brief summary of those arguments.

An optimal value of $T_{\mathrm{ime}}$ will have to satisfy four criteria: (1) it should maximize the metabolic fitness of the animal, through (2) reliable estimation of the past reward rate and temporal delays in the environment (larger the value of $T_{\mathrm{ime}}$, better the accuracy in time perception) so as to (3) appropriately estimate the opportunity cost involved in decisions, while (4) minimizing computational/memory costs. The essence of this optimization is the trade-off between maximizing reward rates and minimizing metabolic costs: integrating over long intervals in stationary environments would lead to better estimates of opportunity costs, but lead to increased metabolic costs associated with the energy spent by the brain in its execution. Further, when environments are nonstationary, the problem becomes even more complicated as one will have to find the duration of history that appropriately represents the current decisions. We will not attempt a quantitative treatment of this problem here. Similar, but less general, optimization problems have been investigated elsewhere (e.g., Behrens et al., 2007; Bialek, 2005; Courville et al., 2006; Nassar et al., 2010; Pearson et al., 2011; Wilson et al., 2013).

We are especially interested in environments that result in low values of the past integration interval, and consequently steep temporal discounting. This is because steep temporal discounting has been associated with a set of behavioral disorders under the umbrella of “impulsivity” (e.g., Evenden, 1999; Madden & Bickel, 2010). Below, we will only focus on the aspects of impulsivity that relate to decisions in the dimension of time.

In a non-stationary environment with a high temporal frequency of instability, the optimal $T_{\mathrm{ime}}$ will have to be correspondingly low so as to provide an appropriate opportunity cost estimate for current decisions. At the other extreme, in a stationary (stationary refers to time-independent statistics and not low variability in the reward statistics) environment in which the variability in reward rate is very low, a low $T_{\mathrm{ime}}$ is sufficient to appropriately estimate the long-term statistics. Hence, here too, since there is diminishing benefits in integrating over longer and longer durations, the metabolic costs will drive down $T_{\mathrm{ime}}$. Another instance where $T_{\mathrm{ime}}$ would be low is when the reward rates are very high. In a highly rewarding environment, any additional benefit in integrating over long intervals would be offset by the increase in metabolic costs. Hence, we predict that individuals living in the above reward environments will discount rewards steeply, because steep discounting is *optimal* in these environments. In this conception, abnormally-steep discounting observed in experiments is not necessarily a sign of a behavioral disorder (viz. impulsivity), but could represent optimality in an individual’s *perceived* reward environment. Of course, this does not mean that every instance of abnormally-steep discounting is optimal; it could also result from aberrations in the brain mechanisms underlying appropriate setting of $T_{\mathrm{ime}}$. Such aberrations might be present in neurobiological disorders leading to impulsive decision-making as seen, for instance, in Parkinson’s disease (Housden et al., 2010; Voon & Dalley, 2011; Voon et al., 2010) and schizophrenia (Avsar et al., 2013; Heerey et al., 2007; Strauss et al., 2014). Interestingly, these disorders are also accompanied with distortions in timing and time perception (e.g., Allman & Meck, 2012; Malapani et al., 1998; Penney et al., 2005).

Since our theory also predicts that abnormally-steep temporal discounting will be correlated with highly non-linear time perception, we would predict, for instance, that in a highly rewarding environment in which $T_{\mathrm{ime}}$ is low, temporal perception would appear sped up. This prediction may underlie anecdotal observations that “*time flies when you’re having fun*”. Our account would also predict that a decrease in reward rate might lead to temporal perception appearing slowed down. This might explain some recent observations (Galtress & Kirkpatrick, 2009; Galtress et al., 2012; Kirkpatrick, 2014) that cannot be easily explained by current timing theories (see Galtress et al., 2012 for a discussion). We would like to point out at this stage that there are other models that also predict a dependence of time perception on the recent history, arguing that recently experienced temporal intervals act as a *prior* for Bayesian optimization of time perception (e.g., Cicchini et al., 2012; Gu & Meck, 2011; Jazayeri & Shadlen, 2010; Shi et al., 2013)

As mentioned in Section 3, variability in $T_{\mathrm{ime}}$ is also expected to underlie 1) deviations from Weber’s law at long durations, and, 2) variations in the point of subjective equality in temporal discrimination tasks. While other models ascribe such observed variability to variability in properties of the accumulator or other neural variables (e.g., Bizo et al., 2006; Killeen et al., 1997), we predict that variability within subjects can arise from a drive to maximize reward rates in varied experimental settings. A strong and unique prediction of this account is that temporal perception is correlated with intertemporal decision-making. Experimental observations have supported this prediction (e.g., Barkley et al., 2001; Barratt, 1983; Bauer, 2001; Baumann & Odum, 2012; Berlin et al., 2004; Berlin & Rolls, 2004; Bickel & Marsch, 2001; Dougherty et al., 2003; Heilbronner & Meck, 2014; Levin et al., 1996; Pine et al., 2010; Reynolds & Schiffbauer, 2004; van den Broek et al., 1992; Wittmann et al., 2007; Wittmann & Paulus, 2008). A stronger, yet untested, falsifiable prediction is that causing changes in the duration over which past reward rate is estimated leads to corresponding changes in discounting steepness and time perception. This is the essence of our theory.

## 5 Conclusion

A rationalization of decision-making in the temporal domain has long been sought, yet an understanding of this problem remains unfulfilled. We have identified three general reasons why there has not yet been significant agreement between theories and experimental observation. The first is that, ever since the first formal attempts to make a reckoning of how humans and animals regard the cost of time, the notion of a discounting function has pervaded, having been ingrained and reified not as a description of observed behavior but rather as a thing with agency applied by the agent to determine an outcome’s subjective value. The second is that intertemporal decision-making has not been framed as reward rate maximization under the constraint of what is feasibly achievable by an agent given the unknowable future. The third is that temporal discounting and time perception have largely been treated as separate problems; when not so, intertemporal decision-making has been regarded as subordinate to time perception rather than being fundamental. These historical biases have combined so that the search for the perfect discounting function, that makes a principled, concise, and full accounting of decision-making in the time domain, has remained an elusive one.

While we posit that, for these reasons, an unresolved tension between theory and observation exists, theories of intertemporal decision-making over nearly two centuries have lead to a sophisticated and rich understanding of this issue, from the recognition of an interplay between the magnitude of an outcome and its cost in time, to its formalization as a psychological conflict, and subsequently to its reframing as a maximization to increase fitness. Nonetheless, theories from economics and behavioral ecology, whether attempting to rationalize intertemporal decision-making within the framework of discounted utility or of reward-rate maximization, fail to provide satisfactory explanations for empirical data, or, in the case of descriptive modeling that provides good fit to observation, fail to provide any normative understanding of why discounting functions take on their apparent form. Recent experiments (e.g., Blanchard et al., 2013; Pearson et al., 2010), however, have shown that what has been previously regarded as clear evidence that animals do not maximize reward rate results from limits in their associative learning.

Recognizing that reward rate maximization could then yet be the fundamental principle behind intertemporal decision-making, we have derived from first principles a decision-making algorithm that would lead to reward-rate maximization under experiential constraints (Namboodiri et al., 2014). In this theory we find that the duration over which the past reward rate is integrated directly determines the tolerance to delays in intertemporal decision-making (i.e. the steepness of temporal discounting) and the non-linearity of time perception. Thereby, we also provide a novel theory of time perception which can explain hallmark behavioral observations (e.g., Weber’s law, point of subjective equality). Unique to our theory, we predict that the ability of individuals to perceive time is correlated with their tolerance to delay in intertemporal decision-making. Therefore, our theory suggests that aberrant timing behavior seen in a range of cognitive/behavioral disorders can be rationalized as a consequence of aberrant integration over experienced reward history. A fundamental test of TIMERR, then, is assessing whether, as the duration over which past reward rates are estimated increases (or decreases), the steepness of temporal discounting decreases (or increases). This is the direct falsifiable test of our theory.
